# Advanced Spatial-Division Multiplexed Measurement Systems Propositions—From Telecommunication to Sensing Applications: A Review

**DOI:** 10.3390/s16091387

**Published:** 2016-08-30

**Authors:** Yi Weng, Ezra Ip, Zhongqi Pan, Ting Wang

**Affiliations:** 1 NEC Laboratories America, Inc., Princeton, NJ 08540, USA; ezra.ip@nec-labs.com (E.I.); ting@nec-labs.com (T.W.); 2 Department of Electrical & Computer Engineering, University of Louisiana at Lafayette, Lafayette, LA 70504, USA; zpan@louisiana.edu

**Keywords:** optical fiber sensors, multiplexing, Brillouin scattering, structural health monitoring, distributed sensors, optical fabrication, birefringence, acoustic wave, fiber Bragg grating, optical time domain reflectrometer (OTDR)

## Abstract

The concepts of spatial-division multiplexing (SDM) technology were first proposed in the telecommunications industry as an indispensable solution to reduce the cost-per-bit of optical fiber transmission. Recently, such spatial channels and modes have been applied in optical sensing applications where the returned echo is analyzed for the collection of essential environmental information. The key advantages of implementing SDM techniques in optical measurement systems include the multi-parameter discriminative capability and accuracy improvement. In this paper, to help readers without a telecommunication background better understand how the SDM-based sensing systems can be incorporated, the crucial components of SDM techniques, such as laser beam shaping, mode generation and conversion, multimode or multicore elements using special fibers and multiplexers are introduced, along with the recent developments in SDM amplifiers, opto-electronic sources and detection units of sensing systems. The examples of SDM-based sensing systems not only include Brillouin optical time-domain reflectometry or Brillouin optical time-domain analysis (BOTDR/BOTDA) using few-mode fibers (FMF) and the multicore fiber (MCF) based integrated fiber Bragg grating (FBG) sensors, but also involve the widely used components with their whole information used in the full multimode constructions, such as the whispering gallery modes for fiber profiling and chemical species measurements, the screw/twisted modes for examining water quality, as well as the optical beam shaping to improve cantilever deflection measurements. Besides, the various applications of SDM sensors, the cost efficiency issue, as well as how these complex mode multiplexing techniques might improve the standard fiber-optic sensor approaches using single-mode fibers (SMF) and photonic crystal fibers (PCF) have also been summarized. Finally, we conclude with a prospective outlook for the opportunities and challenges of SDM technologies in optical sensing industry.

## 1. Introduction

### 1.1. Background Introduction

From the perception of power consumption per bit, the logarithmical channel capacity scaling of up-to-date wavelength-division multiplexed (WDM) coherent optical communication systems has exhausted nearly all possible degrees of freedom, including time, frequency, polarization and phase in single-mode fibers (SMF), and thus can no longer satisfy the ever-increasing demand of exponential global traffic growth [1,2]. To accomplish a further cost-effective scaling in system capacity, space-division multiplexing (SDM) has been proposed as a new paradigm for optical fiber communication research which has attracted loads of attention in the past few years [3,4]. These SDM technologies allow autonomous data streams to be transmitted in parallel spatial channels, which primarily include core multiplexing using multicore fibers (MCF) with a single-strand of fiber with many independent cores to pass through, and mode-division multiplexing (MDM) using multimode fibers (MMF) or few-mode fibers (FMF) whereas one single-core large-area fiber allows a number of spatial guiding modes to travel inside [5,6]. In the intervening time, the optical fiber sensors have been extensively developed owing to their outstanding advantages of high reliability and compactness over the past few decades, whereas SMFs and photonic crystal fibers (PCF) were commonly deployed [7]. Recently SDM-based fiber-optic sensors have attracted broad attentiveness attributable to their potentially higher capacities, sensitivity and flexibilities, compared with conventional SMF-based sensors, via exploring the fifth dimension—the space dimension [8]. Besides, the definition of SDM not only include the spatial mode information in FMF or MCF, but also involve the widely used components with their whole information used in the full multimode constructions, such as the whispering gallery modes for fiber profiling and chemical species measurements, the screw/twisted modes for examining water quality, as well as the optical beam shaping to improve cantilever deflection measurements [9,10,11].

### 1.2. Spatial Division of Information Models for Fiber-Optic Sensing Components

This subsection introduces the possible spatial division of information models fit for use in fiber-optic sensing components. Please note that, although the concepts of spatial channels and modes were first utilized in the telecommunications industry as an indispensable solution to reduce the cost-per-bit of optical fiber transmission, these valuable concepts have been explored recently in other areas of science and engineering, from a fundamental principle point of view [12,13]. Particularly, the spatial modes are applied in fiber-optic sensing applications, as high-speed illuminating signals containing fast-varying data distribution, with the returned echo is then analyzed for the collection of essential environmental information [14,15].

To begin with, the transverse field of linearly polarized (LP) modes in the fiber core *E* (*r*, *θ*) is given by [16]:

(1) $$E\left(r,\theta \right)=E\left(a\right)\cdot \left[\frac{J_{P}\left(k_{r}r\right)}{J_{P}\left(k_{r}a\right)}\right]\cdot \cos\left(p\theta \right).$$

where *r* and *θ* denote polar coordinates, *J_P_* symbolizes the Jones vector, *a* stands for the fiber core radius, *p* represents the azimuthal mode number. The non-negative integer *k_r_* can be expressed as [17]:

(2) $$k_{r}={\left(k^{2}{n_{Core}}^{2}-\beta^{2}\right)}^{\frac{1}{2}}.$$

where *n_Core_* is the refractive index of the fiber core, which determines the material dispersion along the fiber. The propagation constant β determines how fast electric vectors are oscillating during propagation through the optical fiber, which can be written as [18]:

(3) $$\beta =\left[\Delta -\Delta \cdot \left(\frac{U^{2}}{V^{2}}\right)+1\right]\cdot n_{Core}\cdot k.$$

where *U* denotes the dimensionless modal number, *V* symbolizes the normalized frequency, k denotes the free-space wave number. The relative refractive index of the fiber Δ is calculated as:

(4) $$\Delta =\left(n_{Core}-n_{Clad}\right)/n_{Core}.$$

where *n_Clad_* denotes the refractive index of the homogeneous cladding. The normalized frequency *V* can be described as [19]:

(5) $$V=\left(\frac{2\pi }{\lambda }\right)\cdot a\cdot {\left({n_{Core}}^{2}-{n_{Clad}}^{2}\right)}^{\frac{1}{2}}.$$

where *λ* stands for the wavelength. Furthermore, the index profile of a graded-index fiber core *n*(*r*) is given by [20]:

(6) $$n\left(r\right)=n_{Core}\cdot {\left[1-2\cdot \Delta \cdot {\left(\frac{r}{a}\right)}^{\alpha }\right]}^{\frac{1}{2}}.$$

where *α* signifies the power coefficient of the graded-index fiber profile.

**Figure 1:**
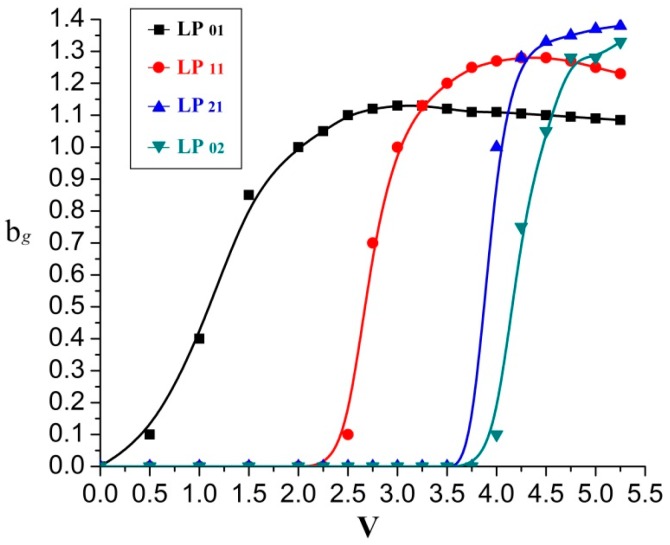
Normalized group delay $b_{g}$ vs. the normalized frequency V.

The amount of guided modes propagating invariant along an optical fiber can be determined by the normalized frequency *V*, as the solution of wave-equation describing an electro-magnetic field distribution [21,22]. The transverse intensity distribution diverges strongly along the FMF, for each spatial mode propagates at a different phase velocity [23,24,25]. However, to excite a single higher-order mode in the fiber, a stable transverse beam pattern is required [26,27]. Figure 1 exemplifies the dependency of the normalized group delay $b_{g}$ upon the normalized frequency V for the first four LP modes, which is expressed as [28]:

(7) $$b_{g}=b_{eff}+\left(\frac{db_{eff}}{dV}\right)\cdot V.$$

**Figure 2:**
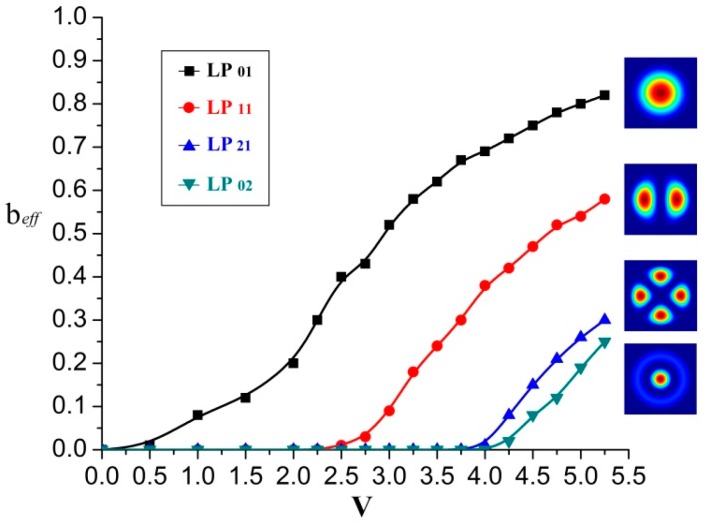
Normalized propagation constant *b_eff_* vs. *V* for LP modes under weakly coupling approximation.

In the meantime the corresponding normalized propagation constant *b_eff_* versus V is shown in Figure 2, which regulates the dispersion properties of various fiber modes as attained in the following equation [29]:

(8) $$b_{eff}=\left[\left(\frac{\beta }{k}\right)-n_{Clad}\right]/\left(n_{Core}\cdot \Delta \right).$$

### 1.3. Benefits of SDM Sensing Systems

In addition to the original advantages of distributed and “smart” fiber-optic sensors, which include the immunity to electromagnetic interference, the avoidance of electric sparks, as well as the resistance to harsh and hazardous environments, the unique benefits of implementing SDM techniques in fiber sensors are summarized in the following.

#### 1.3.1. Measuring More Parameters

In conventional SMF-based distributed fiber-optic sensors, each technique is often applied to the measurement of one single parameter, because each of the parallel sensing signals requires a separate channel. For instance, distributed temperature sensing (DTS) system based on Raman scattering is only dedicated to determine local temperature, while distributed acoustic sensing (DAS) system based on Rayleigh scattering mostly provides strain determinations [30]. To break this bottleneck and further extend the functionality of distributed fiber-optic sensors, the SDM techniques have been introduced for the capability of responding to a wide variety of measurands simultaneously, for each of the modes or cores within the sensing medium can serve as an orthogonal interrogator or geophone for one particular sensing parameter. For example, as mentioned below, when minimum two spatial modes are used to separate the strain and temperature variations, the rest of modes can be further utilized to monitor other physical changes such as pressure, displacement, acceleration, etc. [31].

#### 1.3.2. Multi-Parameter Discriminative Capability

As for the multi-parameter discrimination issue, SMF similarly has its own limitation. For instance, the most common method using a SMF is to measure both the Brillouin frequency shift (BFS) and the Brillouin power level, as Brillouin power is also related to strain and temperature. The different dependencies of the BFS peaks are calculated to distinguish between temperature and strain. Nevertheless, the measuring range and resolution of this method are mainly limited by the imprecise Brillouin power measurement [32]. In another SMF approach, both Raman and Brillouin signals are spatially resolved to separate temperature and strain. The magnitude of anti-Stokes Raman signal intensity determines the temperature, while the strain can then be computed from BFS. However, noise arises mainly from the Raman intensity measurement. Besides, this approach requires both direct detection and coherent detection system components that add additional cost and complexity to the sensing system [33]. Other groups proposed to utilize large effective-area fiber (LEAF) to achieve simultaneous temperature and strain sensing, which creates multiple BFSs within one single fiber core. Nonetheless, this approach leads to poor spatial resolution, limited sensing accuracy and short sensing distance due to large interference between different wavelengths [34]. Henceforward, the above-mentioned problems can be resolved using SDM techniques, because as explained below, each spatial mode or core possesses a unique Brillouin gain spectrum (BGS) or BFS, with which temperature, strain or other parameters can be accurately separated by solving a set of simultaneous equations. Such exceptional multi-parameter discriminative capability is actually contingent on the correlation in-between spatial modes and/or cores as parallel sensors, with various parameters separated using massive multi-input multi-output digital signal processing (MIMO-DSP) solutions [35].

#### 1.3.3. Accuracy Improvement

Another key advantage of SDM techniques is focused on the accurate detection of the backscattered signal and elimination of noise. The conventional SMF techniques are not effective in reducing the coherent Rayleigh noise (CRN) or fading noise [36]. Since FMF or MCF has a rather short coherence time and coherence length, so the superposition will be incoherent and thus CRN negligible. The noise can be further eliminated by using the frequency shift averaging (FSAV) techniques [37]. Since SDM-based sensors have the ability to be easily multiplexed with digital signal processing to determine the positional variation of the measured field along the interaction length with different groups of modes, two or more different spatial modes can be used to do error correction upon the same channel, thus enabling fiber sensing systems capable of performing much more sophisticated and multifunctional types of measurements with higher spectral resolution and faster time response that previously were only achievable using electronic sensors [38]. For example, with six spatial modes, at most three modes can be applied to measure the temperature, and the other three modes for monitoring the strain. This is analogous to having three independent SMFs determining one physical change, respectively, which will significantly improve the temperature and/or strain measurement accuracy.

#### 1.3.4. Detection Speed Enhancement

Detection speed is another important performance aspect for industrial sensing applications, such as oil and gas production monitoring [30,39]. As we know, finding oil leaks does little good if it takes more than several minutes of computer processing to identify them. In SDM measurement systems, the overall number of parallel channels is largely increased with each independent channel on an orthogonal spatial mode, thus enabling higher data rate and real-time sensing for a variety of applications such as well integrity monitoring and down-hole seismic acquisition [40]. For instance, compared to the conventional time-consuming SMF techniques, where several different types of lasers are prepared and alternatively used in order to compare their performances like the hybrid Raman-Brillouin sensing, the SDM approach can simply use two or more spatial modes to simultaneously measure strain and temperature, reducing the process time to about 30 seconds and making the whole process real-time monitoring [39,41].

In this paper, the recent progress in SDM-based sensing systems is reviewed in terms of their operation principles, fabrication methods, experimental design and sensing applications. The outline of the paper is laid out as follows: it starts by introducing the principal components of SDM techniques in Section 2, comprising laser beam shaping, mode conversion, multiplexers, multicore head of sensor elements using LPG and other specific fibers, SDM amplifiers and EDFAs, as well as the detection units of SDM measurement systems. Section 3 describes different examples of SDM-based sensor techniques, including Brillouin optical time-domain reflectometry/Brillouin optical time-domain analysis (BOTDR/BOTDA) using FMF, as well as the MCF-based integrated fiber Bragg grating (FBG) sensors. Section 4 presents the overall summary, comparison and concluding remarks of this paper, dedicated to provide a prospective outlook for the opportunities and challenges of SDM sensing technologies for various markets and applications.

## 2. Key Components of SDM Technique

As discussed in Section 1, the SDM-based optical measurement systems may provide discriminative capability, higher sensitivity and flexibilities, while keeping the fabrication cost at a relatively low level. Therefore, it is critical to design and fabricate corresponding SDM components with proper modal properties to support these novel sensing systems. This section illuminates a number of essential SDM components, such as laser beam shaping devices, mode convertors, multiplexers, amplifiers. It also gives a brief discussion on multicore head of sensor elements, the detection units of SDM measurement systems and the corresponding signal processing algorithms.

### 2.1. Laser Beam Shaping

**Figure 3:**
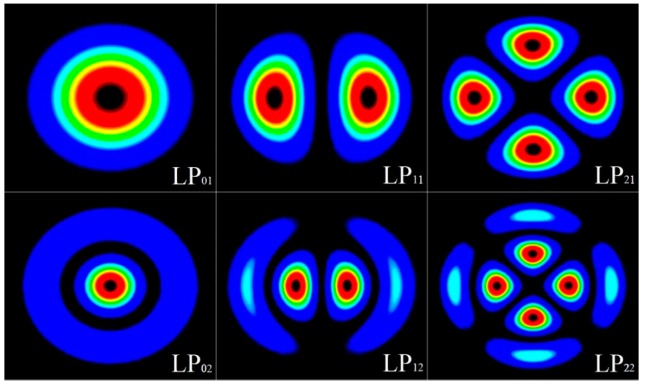
Intensity profile distributions of ideal LP spatial modes.

The key techniques of SDM reply on the rapid development of efficient and automated spatial filtering designs, which converts the input Gaussian beams into desirable outputs via laser beam shaping approaches [42,43]. With an all-fiber beam shaper or similar optical device, the concave cone tip at the end face of single mode and multimode fibers can be inverse etched, so that the light beam can be reshaped with its spatial properties modified [44,45]. These techniques offer extensive coverage of practical laser beam shaping applications such as mode converters and spatial multiplexers/de-multiplexers [46]. The intensity profile distributions of the first six ideal LP spatial modes are shown in Figure 3. They are shaped due to slightly dissimilar propagation constants between the vector modes, resulting in the cross-sectional intensity pattern rotation of LP modes along the optical fiber, whereas the elliptic boundary specifies the core-cladding interface [47].

Besides the most commonly used LP modes, the other types of spatial modes applied in SDM systems include the supermodes, principle modes, transverse modes, screw/twisted modes, whispering gallery modes, as well as the modes of capillary optical fibers [48]. The so-called supermodes indicate the different scale of power transfer between cores in MCF [49]; while to reduce the negative impact of modal dispersion in FMF, the principle modes stand for a basis of spatial modes which are free of modal dispersion to the first order in frequency [50,51]. The transverse modes, including both the transverse electric (TE) and transverse magnetic (TM) polarization modes, are more fundamental propagation modes with their electric and magnetic field lines restricted to directions normal to the direction of modal propagation, whereas complex spatial filters can be implemented by diffractive optical elements corresponding to rotationally symmetrical transverse modes [52]. The screw/twisted modes include the celebrated orbital angular momentum (OAM) states or vortex modes, which have a variety of applications from atmospheric turbulence monitoring, lateral motion detecting, and biomedical image sensing [53,54,55]. Whispering gallery modes are shaped by microscopic glass spheres from the micro-cavities of resonant optical sensors, which can travel around concave surfaces for the applications of frequency-comb generation, opto-mechanical cooling as well as chemical species sensing [56,57]. Last but not least, the modes of micro-structured capillary optical fibers include LP modes, TE modes, and TM modes with low modal confinement losses and group velocity dispersion, shaped by capillaries filled narrowly inside round cavities [58,59].

### 2.2. Mode Generation and Conversion

The next issue would be spatial mode generation and conversion for SDM systems. To begin with, the LP modes can be converted by imposing spatially varying modulation upon the laser beams via variable phase/amplitude masks [60,61]. For instance, spatial light modulators (SLM) is capable of transforming a fundamental LP_01_ mode into higher order modes using liquid crystal on silicon (LCoS) panels or thin phase plates with prescribed spatial distributions of refractive index [62,63]. Also, higher-order LP modes could be generated using fiber Bragg grating, fused fiber coupler, as well as intermodal four-wave mixing [64,65,66]. Similarly, supermodes, principle modes and transverse modes can be rehabilitated via an optically induced long-period grating (LPG) or thin phase plates [67,68,69]. The flexible conversion among multiple OAM modes or vortex modes can be realized by the cylindrical lenses or the helical gratings (HGs) with both transverse and longitudinal modulation, or by an optical parametric oscillator based on cavity and anisotropy effects [70,71]. The whispering gallery modes can usually be configured through a whispering gallery mode resonator in a tapered fiber with low scattering-loss and easy alignment, while the modes of capillary optical fibers can be shaped and changed using a capillary tapered mode converter filled inside round cavities [72,73].

### 2.3. Multiplexers and De-Multiplexers

**Figure 4:**
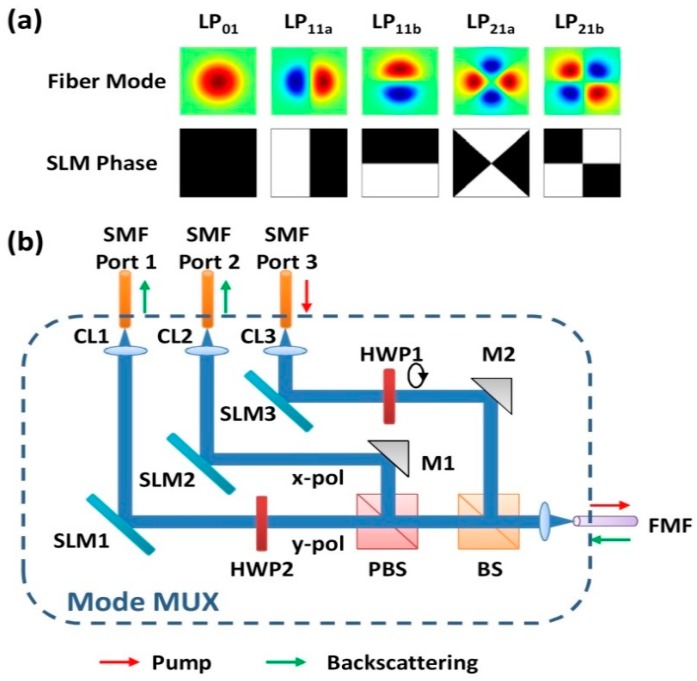
(**a**) Intensity distributions of the first five spatial modes in FMF and their corresponding SLM phase patterns; (**b**) Schematic diagram of the free-space SLM-based M-MUX. CL1~CL3: collimating lens, M1/M2: turning mirrors, HWP1/HWP2: half wave-plates, BS: beam-splitter, PBS: polarizing beam-splitter [77].

A mode multiplexer (M-MUX) can launch all the spatial modes into the FMFs or MCFs, whereas the spatial light modulators (SLM) based on liquid crystal on silicon (LCoS) can convert the fundamental mode in the SMF into arbitrary desirable spatial modes [74,75,76]. The typical configuration of an M-MUX is presented in Figure 4, which uses three programmable SLMs to generate specific LP modes [77]. The relationship between the generated spatial modes in the fiber and their corresponding SLM phase patterns is provided in Figure 4a. In Figure 4b, SLM3 is set for launching the pump power into a spatial mode, SLM1 and SLM2 are configured for the detection of back-scattered light in separable modes, while two half-wave-plates (HWPs) are placed after SLM1 and SLM3 to switch each state of polarization. This M-MUX may also serve as a mode de-multiplexer (M-DMUX) if the beam is launched thru the opposite direction. Furthermore, in order to further reduce the passive multiplexing loss, the photonic lantern (PL) is purposed to transfer the transverse field into super-modes of a three-coupled-core fiber, and then into the FMF mode profiles using an integrated-optics embodiment of the spot coupler [78,79].

### 2.4. Multicore Elements Using Special Fibers

The SDM sensing technologies can be further advanced via multi-core approaches to achieve better system scalability and flexibility along with combination of multi-level modulation. This subsection mainly describes the multi-core elements for addressing individual cores in optical sensors. For specialty sensing MCFs with enhanced spatial-channel densities, by using adhesive and heating up the fiber bundle in the capillary, a more compact configuration with fiber cores fixed in the capillary can be achieved compared to just a bundle of SMFs [80]. When the light is introduced into the cores of MCFs by separate light sources, the alignment of tapered MCF and SMF can be used to control the division of light power among the non-uniformly distributed fiber cores in the cross-section of MCF [81]. Since there is no overlapping between modes propagating in the discrete cores of MCF, each with identical transverse-mode profiles, the MCF-based SDM measurement systems are assumed hypothetically to be with ignorable loss at multiplexers/de-multiplexers, thus provide substantial improvements in the signal-to-noise ratio of backscattered sensing signals [82]. One possible drawback could be the additional complexity and cost of transmitting and detecting signals from different cores to accomplish simultaneous measurements. Thanks to the stack and draw procedure generally used for the PCF fabrication, the multi-core elements can be manufactured at a relatively acceptable price.

### 2.5. Multicore Head of Sensor Elements Using LPG and Other Specific Fibers

As a crucial component of MCF-based SDM sensing systems, the multicore head of sensing elements can be fabricated based on the amorphous wire magneto-impedance elements in combination with a complementary metal-oxide-semiconductor (CMOS) pulse sensor circuit [83]. Such design can be used to develop a highly sensitive magneto-impedance sensor with low noise and stable pico-tesla resolution. Another way to design such multicore head of sensing elements is to UV-inscribe a long period grating (LPG) into MCF, which is fusion spliced into SMFs at both ends [84]. Such configuration leads to a taper transition between MCF and SMF, and creates a non-adiabatic mode evolution, whose spectral characteristics can be used for highly sensitive curvature sensing applications [85].

The MCF-based SDM sensing systems can be employed for a variety of sensing applications. For example, the bending radius as well as the orientation of bending plane can be measured. The bending direction and the amount of deflection can be detected by the outer cores, while the center core provides the reference level for it has the lowest bending loss in the middle of the cross-section of MCF [86]. Besides bend/shape sensing, another significant sensing application of MCF-based SDM measurement systems is simultaneous multi-parameter sensing by utilizing one single optical fiber, since each core can be designed sensitive to different external factor such as strain, temperature or pressure. For instance, the phase shift of far-field interferometric grid-pattern can be generated as a function of curvature, twisting angle and temperature gradient by a four-beam interferometer using MCF for prospective applications in smart structural health monitoring [87].

### 2.6. Single-Core Multimode Elements as Asymmetrical Coupler

Moreover, single-core multimode elements as asymmetrical coupler serve as a significant optical component in SDM-based measuring systems, because the efficiency of the asymmetrical coupler determines the power budget and the quality of optical signal passed to the detection unit, thus affecting the overall sensitivity of detection block [88]. Such asymmetrical coupler can be fabricated via a fusion-tapering technique by stripping off the polyethylene jacket and gluing upon a glass substrate so as to allow maximum multi-mode signal extraction [89]. The constructional parameters of thus-designed asymmetrical coupler include the length/depth of the coupling area as well as the curvature radius, whereas the sensor head is mounted on a mini-lift and formed by the end of a large-core polymer optical fiber [90].

### 2.7. SDM Amplifiers and EDFAs

Inline amplifiers as Erbium-doped fiber amplifiers (EDFA) or Raman amplifiers are sometimes used in measurement systems where the signals are weak [91,92]. If these measurement systems are extended to the spatial domain, the overall system performance could be hindered by the use of conventional amplifiers, because each spatial mode experiences a different value of optical gain due to distinctive field profile configurations [93]. Therefore, to extend sensing reach and achieve stable performance of SDM-based measuring systems, the design of SDM amplifiers would be hypothetically essential to the process.

**Figure 5:**
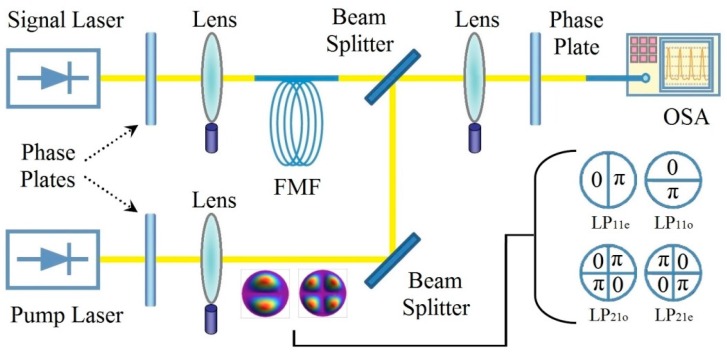
Schematic of the few-mode distributed Raman amplifier.

Up to now, various multimode amplification approaches have been purposed towards the improvement of mode-dependent gain [94]. Though most of them were still mainly intended for transmission purposes, these amplifiers have great potential in various sensing and network application, for instance, to achieve ultra-long distance sensing systems [95]. Firstly, few-mode erbium-doped fiber amplifier (FM-EDFA) has been purposed to equally and efficiently amplify both modes and wavelengths over the target band as a balance between differential group delay, noise figure, crosstalk and cost efficiency [96]. It has been experimentally demonstrated that by adjusting pump mode orientation, higher modal gain and favorable gain equalization can be realized for FM-EDFA, while its major challenges include the signal power fluctuations due to random mode coupling (RMC) [97]. On the other hand, to tune the modal dependent gain over a dynamic range, few-mode Raman amplifier serves as a favorable alternative for SDM systems in comparison with FM-EDFAs [98]. The few-mode Raman amplifier may accomplish a substantial improvement in the noise performance, for the distributed nature of Raman amplification allows a lower input signal power [99]. The typical configuration of few-mode Raman amplifier is schematized in Figure 5 whereas the LP_11o/e_ and LP_21o/e_ tributaries are also shown in the inset. The signal and pump lead through the SLMs to convert to higher-order modes, while the modal dependence of gain or noise figure at each output port of M-DMUX could be measured by an optical spectrum analyzer (OSA) [100].

Moreover, multicore fiber amplifiers have also been designed and constructed for amplifying SDM signals, in order to minimize the noise figure while achieving large gain and broad band width, which is dependent on the overlap integral of the cladding-guided pump field and the doped cores [101,102]. Hypothetically, a FM-EDFA should be slightly more cost-efficient than a multi-core EDFA thanks to its denser spatial packing of multiple channels [103].

### 2.8. Opto-Electronic Sources and Detection Units of Sensing Systems

This subsection describes a number of detectors and opto-electronic light sources for SDM measurement systems. The opto-electronic sources mainly include distributed feedback laser diodes (DFB lasers), vertical-cavity surface-emitting lasers (VCSEL), Fabry-Perot laser diodes (FP lasers), Nd:YAG lasers, and quantum cascade lasers (QCLs) [104,105]. The development of SDM-based sensing systems is pushing the boundaries of high-speed multi-wavelength opto-electronic sources and modules, making other kinds of low-cost light sources possible for SDM. For instance, such opto-electronic sources can be integrated on the silicon platform on GaAs, Si and InP, etc. [106,107].

The detection units can be divided into two groups, direct detectors and coherent detectors. High-efficient direct detection can be achieved with avalanche-photodiode-array (APD-array) by adopting appropriate modulation and multiplexing techniques [108]. Compared with non-coherent direct-detection receivers, coherent receivers have plentiful advantages including remarkably improved selectivity and sensitivity, at the cost of higher computational complexity [109,110,111]. In digital coherent detection, both in-phase (I) and quadrature (Q) components of optical signals from different mode channels are coherently and synchronously digitized using a carrier phase reference generated at the receiver, and then processed using digital signal processing (DSP), for the mode coupling in the fiber is sensitive to the phase of the signals [112,113]. The coherent detection can be implemented using either homodyne detection or heterodyne detection. As a general rule, the homodyne approach involves a bandwidth on the level of the symbol rate with two balanced receivers; meanwhile heterodyne needs one balanced optical receiver with twice the electrical bandwidth [114].

**Figure 6:**
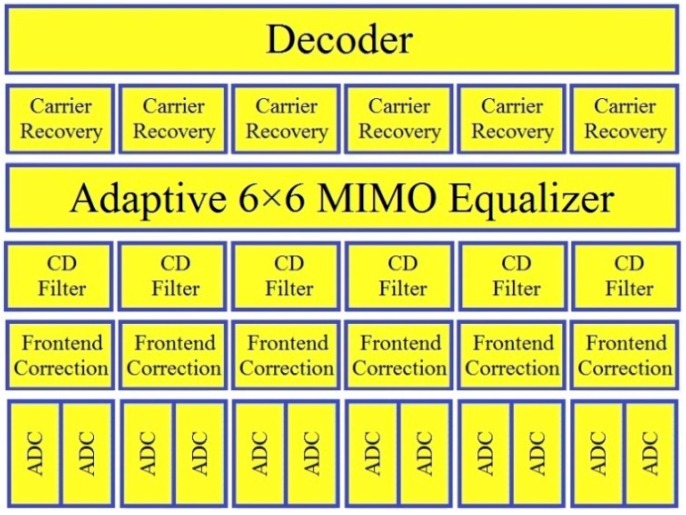
Typical DSP architecture for the MIMO equalization. ADC: analog-to-digital converter; CD: chromatic dispersion.

In SDM sensing systems, since mode coupling might occur between the spatial modes induced by M-MUX/M-DMUX and/or FMF, multiple-input-multiple-output (MIMO) DSP is usually prerequisite to de-multiplex the signals on different modes and dynamically compensate much impairment in the electric domain [115,116,117]. Figure 6 illustrates the coherent receiver structure for a single carrier system with 6 × 6 MIMO scheme, whereas the coefficient adaptation could be achieved with the decision-directed least mean square (LMS) algorithm [118,119]. Such multiplexing techniques can achieve better signal-to-noise ratio (SNR) while compared with complementary decoding, thus suitable for distributed sensing applications [120,121].

## 3. Examples of SDM Based Sensing Systems

As described earlier, the general maturing of SDM components leads to the potential of developing a highly sensitive and stable optical sensing system for multi-parameter sensing with discrimination capability, which is suited to structural health monitoring (SHM) systems in harsh environment applications, such as temperature and/or pressure sensing for the petroleum industry [122,123]. In this section, numerous types of SDM-based sensing techniques are explored, such as distributed sensors using FMFs and/or MCFs, and discrete sensors based on Fiber Bragg grating (FBG). Besides, the whispering gallery modes for fiber profiling and chemical species measurements, the screw/twisted modes for examining water quality, and the optical beam shaping to improve cantilever deflection measurements are discussed as well.

### 3.1. Distributed Sensors Based on Mode-Division Multiplexing (MDM)

To achieve advanced flexibilities and sensitivity over conventional SMF-based approaches, in recent times there has been an emergent interest in developing FMF-based optical sensors, whilst maintaining the fabrication cost at a comparatively low level [124]. In this subsection, few-mode fiber-optic sensors are evaluated in terms of operation principle, fabrication approaches, experimental design and sensing applications.

#### 3.1.1. Operation Principle

There are a number of fiber-optic distributed sensing techniques that rely on three different scattering mechanisms including Raman, Brillouin and Rayleigh scattering, amongst which Raman and Rayleigh could not fully provide information of temperature and/or strain distribution, because Raman scattering is only related to temperature, while Rayleigh scattering has no Stokes/anti-Stokes waves [125]. In contrast, Brillouin scattering serves as a useful tool for the distributed temperature and/or strain measurements, which has been intensively studied for SMFs in the past few decades [126]. Since numerous spatial modes are involved in FMFs, the stimulated Brillouin scattering (SBS) could occur not only within the same fundamental mode, but between different modes as well.

**Figure 7:**
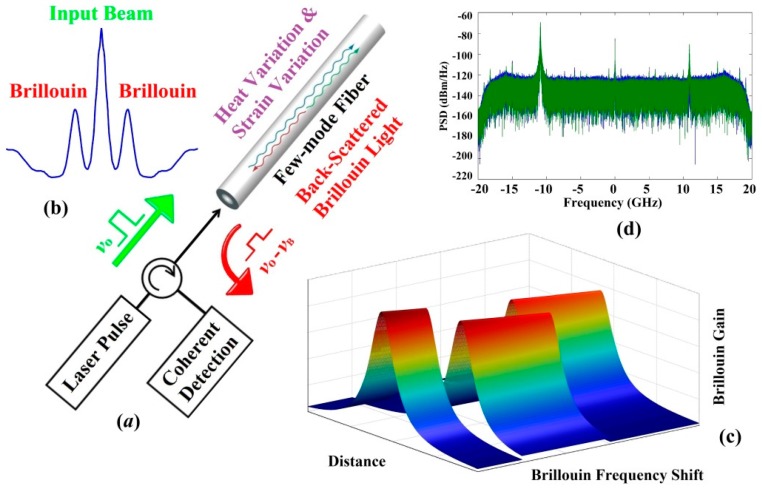
(**a**) Operational principle of optical sensing systems using FMF; (**b**) Schematic of the Brillouin frequency shifts; (**c**) 3D Brillouin gain spectrum with the temperature and/or strain variations; (**d**) Experimental Brillouin spectrum example for LP_01/11_ modes.

The schematic of a few-mode optical sensing system is presented in Figure 7a with $\upsilon_{B}$ and $\upsilon_{o}$ as the altered and reference Brillouin frequency shifts (BFS) correspondingly, whereas the Brillouin scattered light is propagating in the opposite direction and shifted by BFS, which is caused by the nonlinear interaction between the incident light and thermally excited acoustic phonons. The scalar wave equation of the optical field can be described by [127]:

(9) $$\frac{d^{2}f_{o}}{dr^{2}}+\frac{1}{r}\cdot \frac{df_{o}}{dr}+{k_{o}}^{2}\cdot \left({n_{o}}^{2}\left(r\right)-{n_{o\text{​}eff}}^{2}\right)\cdot f_{o}=0.$$

where $f_{o}$ signifies the optical field distribution as a function of radial position r; the subscript o indicates the optical field; $n_{o}\left(r\right)$ symbolizes the optical refractive index for the fundamental mode; $n_{o\;eff}$ represents the effective refractive index of the optical guided modes, while $k_{o}$ denotes the optical wave number related to the optical wavelength λ by 2π/λ.

As displayed in Figure 7b, the back-scattering spectrum after Lorentzian fitting is symmetrical around the incident frequency, and the newly generated peaks are equally spaced by the BFS, which is proportional to both temperature and strain variations. Since it’s theoretically impossible to separate these two effects by only measuring one BFS, lots of methods have been proposed to achieve multi-parameter sensing with discrimination capability [128]. Nonetheless, most early approaches using SMFs either led to poor sensing accuracy or added extra noise and complexity to the system [129]. For the meantime, FMF-based sensors serve as a promising candidate to resolve this issue, because each spatial mode in FMF may have different Brillouin properties, so more than one BFS can be provided to simultaneously discriminate the alterations occurred in temperature and/or strain applied to the optical fiber. To compare the principle of FMF-based sensors with SMF-based counterparts, the BFS of spatial mode one and mode two, $\Delta {\nu_{B}}^{Mode\;1}$ and $\Delta {\nu_{B}}^{Mode\;2}$, are associated with the temperature change ΔT and the strain variation Δε by the following equations [130]:

(10) $$\left(\begin{array}{c} \Delta {\nu_{B}}^{Mode\;1}\\ \Delta {\nu_{B}}^{Mode\;2} \end{array}\right)=\left(\begin{array}{cc} {C_{\nu \;T}}^{Mode\;1} & {C_{\nu \;\varepsilon }}^{Mode\;1}\\ {C_{\nu \;T}}^{Mode\;2} & {C_{\nu \;\varepsilon }}^{Mode\;2} \end{array}\right)\cdot \left(\begin{array}{c} \Delta T\\ \Delta \varepsilon \end{array}\right).$$

Thus the temperature change ∆*T* can be expressed as:

(11) $$\Delta T=\frac{{C_{\nu \;\varepsilon }}^{Mode\;2}\cdot \Delta {\nu_{B}}^{Mode\;1}-{C_{\nu \;\varepsilon }}^{Mode\;1}\cdot \Delta {\nu_{B}}^{Mode\;2}}{{C_{\nu \;\varepsilon }}^{Mode\;2}\cdot {C_{\nu \;T}}^{Mode\;1}-{C_{\nu \;\varepsilon }}^{Mode\;1}\cdot {C_{\nu \;T}}^{Mode\;2}}.$$

Meanwhile the strain variation ∆*ε* is of the form:

(12) $$\Delta \varepsilon =\frac{{C_{\nu \;T}}^{Mode\;2}\cdot \Delta {\nu_{B}}^{Mode\;1}-{C_{\nu \;T}}^{Mode\;1}\cdot \Delta {\nu_{B}}^{Mode\;2}}{{C_{\nu \;T}}^{Mode\;2}\cdot {C_{\nu \;\varepsilon }}^{Mode\;1}-{C_{\nu \;T}}^{Mode\;1}\cdot {C_{\nu \;\varepsilon }}^{Mode\;2}}.$$

Hence, the strain and temperature effects can be discriminated by solving the simultaneous equations, and hereafter attains the sensing information along the FMF. In addition, the Brillouin gain spectrum (BGS) is shown in Figure 7c, while the experimental Brillouin backscatter spectrum is presented in Figure 7d.

**Figure 8:**
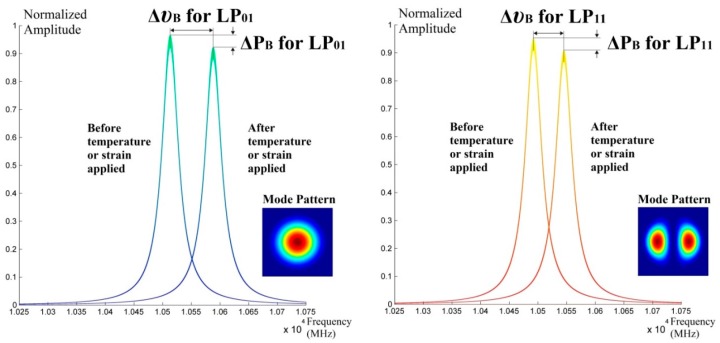
Brillouin Gain Spectra for LP_01/11_ modes in FMF.

The BGS in a FMF that supports LP_01_ and LP_11_ mode are shown in Figure 8, whereas ΔP_B_ denotes the power-level difference of Brillouin scattered light caused by strain or temperature applied to FMF.

#### 3.1.2. Fabrication Methods

**Figure 9:**
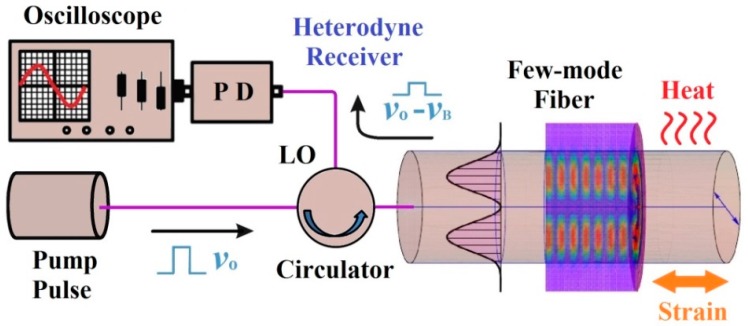
Fabrication and Characterization of a few-mode Brillouin sensing system.

The fabrication and characterization of a few-mode Brillouin sensing system is illustrated in Figure 9. When the incident light propagates through a FMF, the thermally excited mechanical vibrations can propagate as guided acoustic modes in the fiber, while Brillouin scattering spontaneously yields either the frequency down-shifted (Stokes) or up-shifted (anti-Stokes) photons, due to the interaction between the acoustic modes on the optical modes. The corresponding BFS $\nu_{B}$ is described as [131]:

(13) $$\nu_{B}=\frac{2n_{o\;eff}}{\lambda }\cdot V_{eff}=\frac{V_{Clad}}{\lambda }\cdot \frac{2n_{o\;eff}}{n_{a\;eff}}.$$

and the effective refractive index of the acoustic guided modes is given by:

(14) $$n_{a\;eff}=\frac{V_{Clad}}{V_{eff}}.$$

where $V_{eff}$ denotes the effective longitudinal velocity, and $V_{Clad}$ signifies the longitudinal acoustic velocity in fiber cladding.

As mentioned above, the intensity of Brillouin scattering depends on the strong correlation between the longitudinal acoustic and optical modes. The normalized modal overlap integral between optical and acoustic fields $I_{u}$ can be defined as [132]:

(15) $$I_{u}=\frac{{\left(\int E_{o}{E_{o}}^{*}{\rho_{u}}^{*}r\;dr\;d\theta \right)}^{2}}{\int {\left(E_{o}{E_{o}}^{*}\right)}^{2}r\;dr\;d\theta \cdot \int \rho \;\rho^{*}r\;dr\;d\theta }.$$

**Figure 10:**
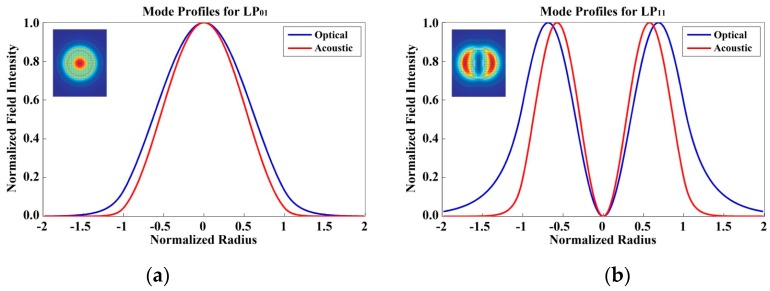
The intensity profiles of optical/acoustic modes for LP_01/11_ in a FMF.

Here the integral brackets denote the integration over the polar coordinates r and θ with the electric field distribution of optical modes $E_{o}$ and acoustic density variation ρ for the acoustic mode of order u. In the meantime, the intensity profiles of optical/acoustic modes in a FMF are illustrated in Figure 10, whereas the optical and acoustic profiles match well for LP_01_ mode, while the overlap integral of optical/acoustic profiles for LP_11_ mode is apparently much smaller. This might elucidate why each spatial mode has slightly different Brillouin property in FMF, and henceforth this overlap integral can be controlled thru the acoustic velocity profile design as well as the fiber refractive index profile design.

#### 3.1.3. Experimental Design

**Figure 11:**
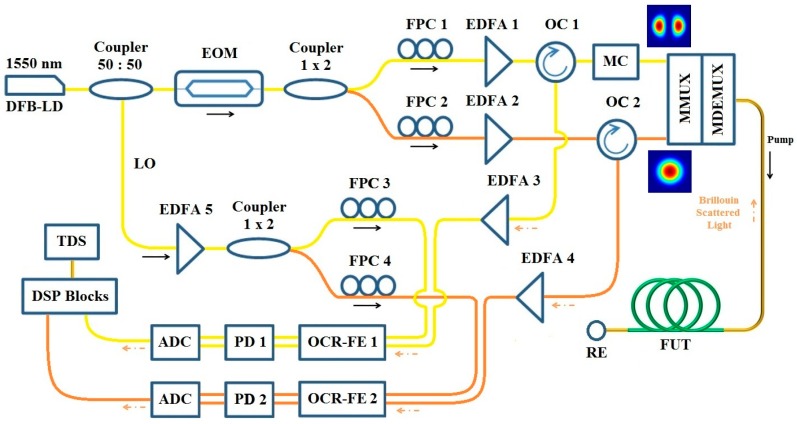
Configuration of BOTDR using FMF for simultaneous temperature and strain sensing. DFB-LD: distributed feedback laser diode; EOM: electro-optic modulator; LO: local oscillators; FPC, fiber polarization controller; OC: optical circulator; FUT: fiber under test; RE: reflective end; OCR-FE: optical coherent receiver front end; PD: photo-detector; TDS: time-domain sampling scope.

The experimental setup of a few-mode BOTDR for simultaneous temperature and strain sensing is depicted in Figure 11. A 1550 nm distributed feedback (DFB) laser diode (LD) is used as a light source, the output of which is divided into two arms by a 50:50 coupler. The pump wave is modulated by an electro-optical modulator (EOM) driven with 30 ns Gaussian pulse to achieve high pump power in the upper path, which is further divided with a 1 × 2 coupler to provide pump power for two different spatial modes. The lower path is amplified by an EDFA, and then divided again by a 1 × 2 coupler to generate two carriers as the local oscillators (LO) for the upcoming heterodyne coherent detection. The fiber polarization controllers (FPC) ensure that they are co-polarized with the pumps. For the upper two paths, another two FPCs and EDFAs are used to control the power and the polarization state of the pumps. Two optical circulators (OC) are inserted before the M-MUX in the pump path, and the mode converter (MC) using phase plate makes sure the pump is launched into any desired higher order LP modes. Then two different spatial modes are mode multiplexed and launched into the fiber under test (FUT). A 4 km circular-core step-index FMF is used as a FUT, with a reflective end (RE) attached to the other side.

The counter-propagating probes are mode de-multiplexed to the original two spatial modes by the same MC component in the pump path. Through two OCs, the optical signal in each mode is re-amplified by EDFAs, and then directed to the optical coherent receiver front end (OCR-FE), which is coupled with a 1550 nm LO from the same light source, whose state of polarization (SOP) is also maintained through FPCs. The coherent receiver includes the optical hybrid and real-time oscilloscope, followed by low-pass filtering, photo-detectors (PDs), ADCs and DSP blocks. The electrical signals of both modes are sampled by a time-domain sampling scope (TDS).

Furthermore, the maximum errors for temperature and strain measurements using FM-BOTDR can be determined in the form of [133]:

(16) $$\delta T=\frac{\left|{C_{\nu \;\varepsilon }}^{Mode\;2}\right|\cdot \delta {\nu_{B}}^{Mode\;1}+\left|{C_{\nu \;\varepsilon }}^{Mode\;1}\right|\cdot \delta {\nu_{B}}^{Mode\;2}}{\left|{C_{\nu \;\varepsilon }}^{Mode\;2}\cdot {C_{\nu \;T}}^{Mode\;1}-{C_{\nu \;\varepsilon }}^{Mode\;1}\cdot {C_{\nu \;T}}^{Mode\;2}\right|}.$$

(17) $$\delta \varepsilon =\frac{\left|{C_{\nu \;T}}^{Mode\;2}\right|\cdot \delta {\nu_{B}}^{Mode\;1}+\left|{C_{\nu \;T}}^{Mode\;1}\right|\cdot \delta {\nu_{B}}^{Mode\;2}}{\left|{C_{\nu \;T}}^{Mode\;2}\cdot {C_{\nu \;\varepsilon }}^{Mode\;1}-{C_{\nu \;T}}^{Mode\;1}\cdot {C_{\nu \;\varepsilon }}^{Mode\;2}\right|}.$$

**Figure 12:**
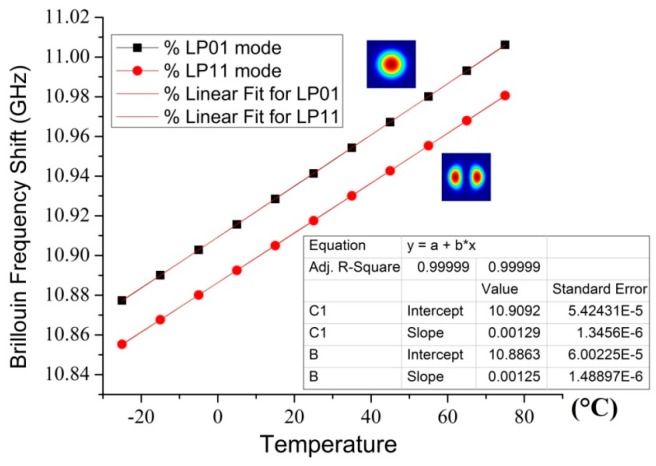
Calibration of temperature coefficients for different modes in FMF.

The BFS dependence of LP_01_ and LP_11_ modes on temperature is shown in Figure 12, when the strain is fixed at 0 *με*, in the inset of which the proportionality coefficients are calibrated, about 1.3 MHz per Celsius degree, via a least squares fitting of linear regression.

**Figure 13:**
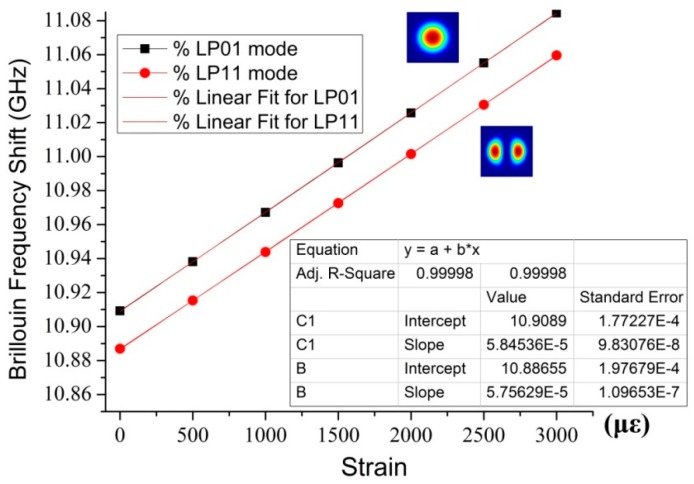
Calibration of strain coefficients for different modes in FMF.

Likewise, the calibrations of the strain proportionality coefficients for different modes in FMF are illustrated in Figure 13, when the temperature is set as 25 °C. By linear regression, the proportionality coefficient is calculated to be around 58 KHz per micro strain.

**Figure 14:**
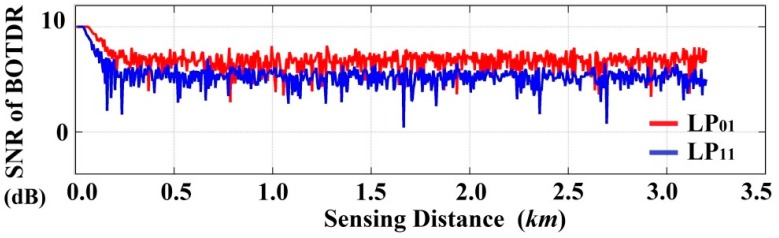
Signal-to-noise ratio (SNR) comparison for FM-BOTDR system between LP_01_ and LP_11_ mode along the sensing fiber.

Furthermore, Figure 14 illustrates the signal-to-noise ratio (SNR) distribution of the few-mode BOTDR system after 20 times averaging, which indicates the LP_01_ mode experiences a bit higher gain over the higher order mode, owing to their dissimilar optical/acoustic correlation profiles. The SNR is expressed as the ratio of maximum and minimum of Lorentzian fitting curve for all the amplitude data at a fixed frequency, whereas the amplitude distribution variability leads to the SNR fluctuations; in the intervening time heterodyne detection has been implemented to increase the system sensitivity, while averaging is performed to enhance the SNR.

In contrast to the proportionality coefficients in standard silica SMF at 1550 nm [128], which are averagely 1.08 MHz/°C and 43 kHz/*με* respectively, both the strain and temperature coefficients (*f*-ε and *f*-T) in FMF are slightly larger, as shown in Table 1, which is caused by the difference of structural deformation in FMF. The LP_01_ mode has slightly larger coefficients, because its intensity profile has stronger correlation between optical and acoustic modes.

#### 3.1.4. Sensing Applications

The distributed MDM sensing systems serve as a novel technique to make simultaneous measurements of both the temporal and spatial behavior utilizing the special properties of FMF as a non-intrusive and dielectric sensing medium. One flexible FMF embedded within the smart structure might substitute thousands of closely attached expensive traditional electronic point sensors, making the distributed sensing system cost efficient [134]. Another key advantage of this technique concentrates on the accurate detection of the backscattered signal as well as the elimination of noise. The conventional SMF techniques are not effective in reducing the coherent Rayleigh noise (CRN) or fading noise. Since FMF has a relatively short coherence length, so the superposition will be incoherent and thus CRN becomes negligible. Coherent detection is necessary for detecting light which propagates with lower and higher order modes, and noise can be further eliminated by using the frequency shift averaging (FSAV) techniques [135]. Thus FMF-based distributed sensors have attracted considerable attention due to their discriminative capability to measure strain and/or temperature, and thus can be applied in a variety of civil and geotechnical structure health monitory (SHM), such as the deformation monitoring and health diagnosis of tunnels, bridges, dams, pipelines, dikes and buildings. Additionally, the MDM sensing systems play an extremely significant role in operation safety for a variety of applications in energy industry, such as well-integrity monitoring and downhole seismic acquisition.

### 3.2. Distributed Sensors Based on Core Multiplexing

An alternative solution to the ever-increasing demand of SDM sensors is based on multicore fibers (MCF). In recent years, novel sensors utilizing MCF have been proposed and demonstrated experimentally for distributed sensing purposes subjected to harsh environments, based on the interference effects in-between the central core and outer cores with longitudinal strain or heat applied to the MCF segment [136].

#### 3.2.1. Operation Principle

**Figure 15:**
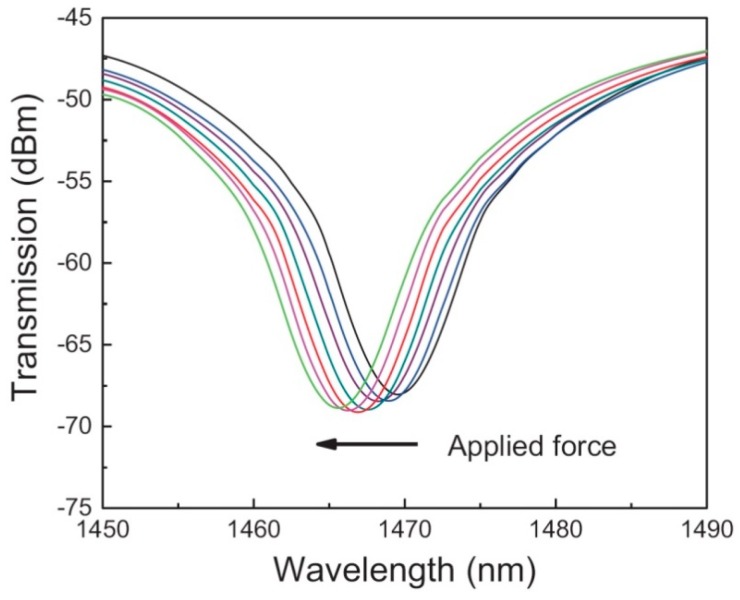
Force-induced wavelength shift of the MCF sensing device [137].

With a broadband light-source at the transmitter side and an OSA at the receiver side, the MCF interference pattern spectrum can be monitored, which would be shifted either by applied strain in keeping with the refractive index changes, or by temperature changes due to the thermo-optic coefficient. The experimental example dips in the transmission spectra of the etched MCF device is shown in Figure 15, whereas different colors of the curves are corresponding to altered magnitudes of applied strain onto the testing MCF [137]. The corresponding Young’s modulus E can be expressed as:

(18) $$E=\frac{\sigma }{\varepsilon }=\frac{F\cdot L}{\Delta L\cdot A}.$$

where σ signifies stress, ε denotes applied strain, F is force, L and ΔL are the fiber length and its change due to the applied strain, and *A* stands for the cross-sectional area.

As mentioned above, the MCF structure has linear responses to both strain ε and temperature T, while the wavelength shift $\Delta \lambda_{MCF}$ can be described as:

(19) $$\Delta \lambda_{MCF}=C_{\varepsilon }\cdot \Delta \varepsilon +C_{T}\cdot \Delta T.$$

where $C_{\varepsilon }$ and $C_{T}$ denote the strain and temperature coefficients derived from elasto-optical coefficient and thermal expansion coefficient of the fiber respectively. Additionally, in order to discriminate the cross sensitivities of two heterogeneous cores in MCF, this correlation can be further expressed in the matrix form as [138]:

(20) $$\left(\begin{array}{c} \Delta \varepsilon \\ \Delta T \end{array}\right)=\left(\begin{array}{cc} {C_{\varepsilon }}^{Core1} & {C_{T}}^{Core1}\\ {C_{\varepsilon }}^{Core2} & {C_{T}}^{Core2} \end{array}\right)\left(\begin{array}{c} \Delta \lambda_{Core1}\\ \Delta \lambda_{Core2} \end{array}\right)=\frac{1}{\det\left(H\right)}\left(\begin{array}{cc} {C_{T}}^{Core2} & -{C_{T}}^{Core1}\\ -{C_{\varepsilon }}^{Core2} & {C_{\varepsilon }}^{Core1} \end{array}\right)\left(\begin{array}{c} \Delta \lambda_{Core1}\\ \Delta \lambda_{Core2} \end{array}\right).$$

where ${C_{\varepsilon }}^{Core\;i}$ and ${C_{T}}^{Core\;i}$ denote the strain and temperature coefficients for the heterogeneous core i in MCF, and det(H) represents the determinant of the coefficient matrix connecting temperature T and strain ε responses with two spatial channels.

#### 3.2.2. Fabrication Methods

**Figure 16:**
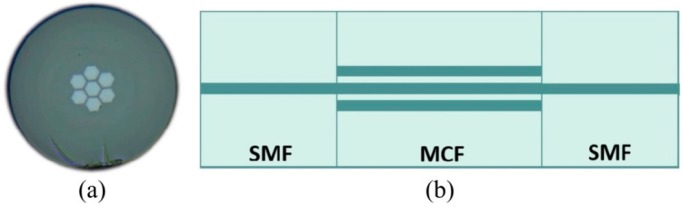
(**a**) Microscope image of a 7-core optical sensing fiber; (**b**) Schematic diagram of MCF-based sensing device structure [138].

The fabricated cross-section of sensing MCF with seven germanium-doped coupled cores inside is shown in Figure 16a, while the configuration of the MCF sensing structure made of one MCF spliced between two SMFs is presented in Figure 16b for simultaneous temperature and force sensing purposes [138].

This etched MCF sensing device was fabricated in-house in 6:1 buffered oxide etch (BOE) at a rate of 0.24 μm/min, with a numerical aperture (NA) of 0.13, a pitch of 12.1 μm, an insertion loss of less than 0.05 dB, with core diameters of 10.6 μm as well as an outer diameter of 125 μm. Besides, the sensing sensitivity of the MCF sensors can be improved by decreasing the fiber outer diameter, for a smaller cross-sectional area leads to a higher average applied force per area in accordance with the Young’s modulus.

#### 3.2.3. Experimental Design

**Figure 17:**
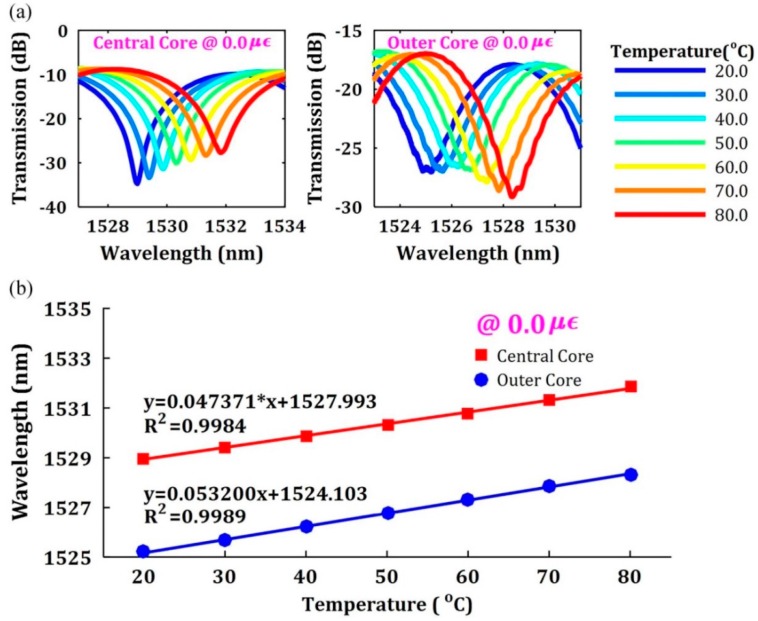
(**a**) Transmission spectrum shift of the central core and the outer core with different temperature; (**b**) Transmission spectrum response as a function of temperature [139].

The spectral responses to temperature of the central core and the outer core in MCF when there is no applied strain are presented in Figure 17a,b, whereas as temperature rises from 20.0 °C to 80.0 °C, the spectrum dips would shift to a longer wavelength with corresponding temperature sensitivities of 47.37 pm/°C and 53.20 pm/°C respectively based on a linear fitting of *R*^2^ values all above 0.998 [139]. Furthermore, the SDM multi-parameter measurement coefficients with discrimination using MCF are summarized in Table 2.

#### 3.2.4. Sensing Applications

Likewise, the distributed fiber sensors based on core multiplexing can be deployed in multiple industrial segments, such as oil and gas production, power cable monitoring, leakage detection at dikes and dams, integrity of liquid natural gas (LNG) carriers and terminals, thanks to its low-cost, superior sensitivity, light-weight, electrical-safety, remote-access, and the ease of being multiplexed [140]. When the minimum two spatial channels are used to separate the applied strain and temperature, the rest of cores can be further utilized to monitor other physical measurand such as pressure, displacement, or acceleration.

### 3.3. Fiber Bragg Grating (FBG) Sensors Based on Core Multiplexing

A novel SDM sensor based on co-located multicore FBGs is introduced in this subsection in terms of operation principle, fabrication methods, experimental design and sensing applications, which provides an estimation of fiber shaping and bending thru measuring distributed fiber curvature for potential applications such as submarine towed-instrument tracking and morphing-wing shape monitoring [141].

#### 3.3.1. Operation Principle

Although the SMF FBG only has one resonant dip in the transmission spectrum, FBGs based on MCF might have more than one resonant dips. By analyzing the changing spectra of the dips, the changes induced by bending, stressing or temperature fluctuations can be distinguished. Different dips have different sensitivities in bending fluctuations, attributable to the difference in structural deformation when strain is applied to the MCF. For similar reasons, the shapes of multiple dips would be impacted by temperature variations in different behaviors [142]. Besides, one of major advantages of grating-based fiber-optic sensors is that they can be simply multiplexed. As each grating is inscribed at different locations on the sensing fiber with different grating periods, the signals coming from each core are encoded at different positions in the wavelength domain. The FBG resonant wavelength depends on the effective index of refraction of the core and the periodicity of the grating, so the shift in MCF FBG center wavelength $\Delta \lambda_{B}$ owing to strain and temperature variations Δε and ΔT can be written as [143]:

(21) $$\Delta \lambda_{B}=\lambda_{B}\left[\left(1-p_{e}\right)\cdot \Delta \varepsilon +\left(\alpha_{\Lambda }+\alpha_{n}\right)\cdot \Delta T+C\right].$$

where $p_{e}$ denotes the effective strain-optic constant, $\alpha_{\Lambda }$ signifies the thermal expansion coefficient for the fiber, while $\alpha_{n}$ represents the thermo-optic coefficient. Last of all, the proportional constant C stands for the FBG wavelength shift caused by other parameters such as pressure, chemical concentration or PH values, etc. Thus, multiple physical quantities can be easily and simultaneously measured by the spectral peak shift in the wavelength range, thru multi-core FBG sensing along the fiber.

#### 3.3.2. Fabrication Methods

**Figure 18:**
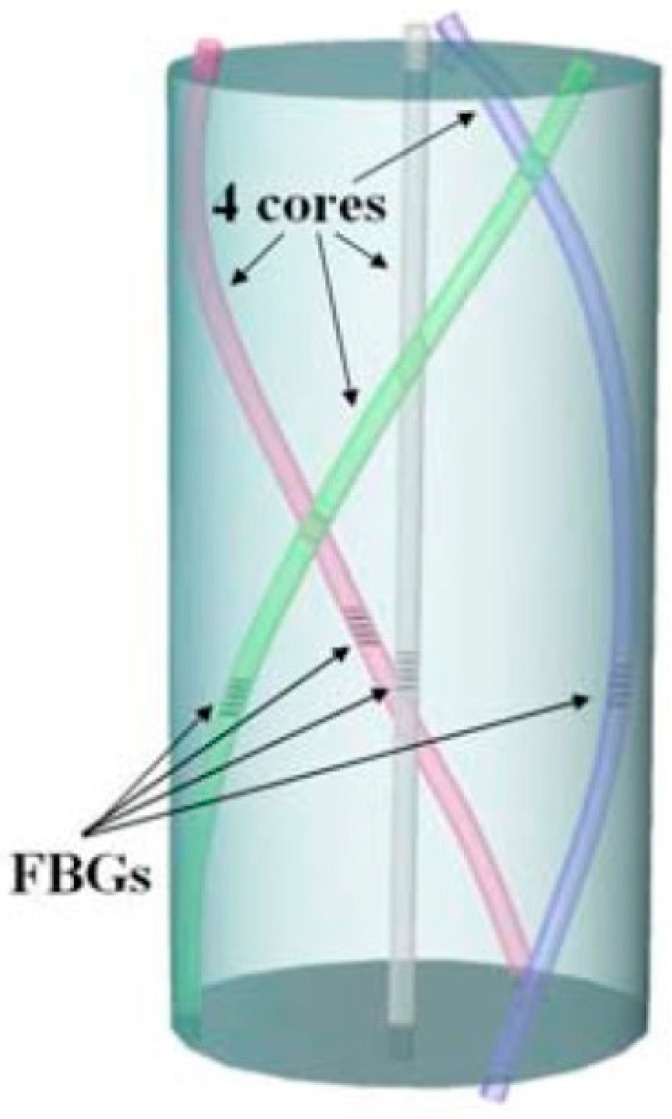
Configuration of four-core twist-biased FBGs [144].

The short segment fabrication of four-core shape-sensing FBGs is displayed in Figure 18, with one nominally on-axis central core as a reference and three extensively displaced outer cores in azimuthal angles for explicit bending sensing purposes, whereas four interrelated temperature or twist-induced strain signals can be detected by means of four-FBG rosettes all aligned at axial coordinate for multi-parameter discrimination [144].

#### 3.3.3. Experimental Design

**Figure 19:**
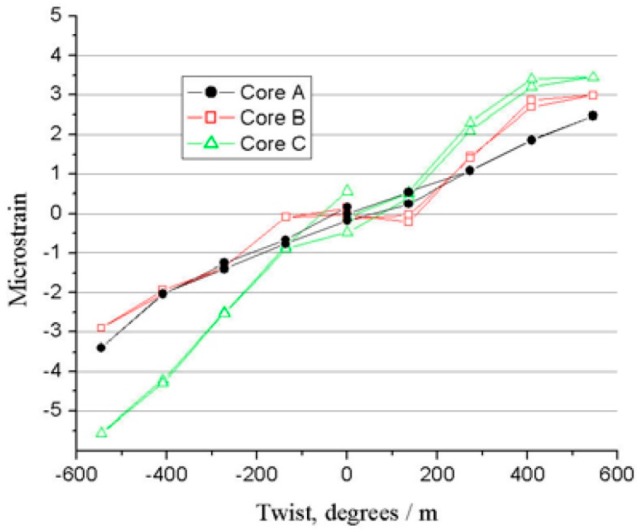
Twist-to-strain response of MCF-FBGs in one rosette [145].

The twist-to-strain response and robust shape prediction within 4-core fiber tethers are tested under low reflectivity using rosette solution algorithms by incorporating twist measurements into the shape elucidation, as shown in Figure 19, whereas the twist coefficients range from 4.8 to 8.9 nε/degree-m in various FBG cores, and the twist accuracy is approximately 50 degrees/m due to imperfections in some fiber cores [145].

#### 3.3.4. Sensing Applications

Thanks to their distinctive filtering properties and adaptability as in-fiber devices, FBGs have been under much attention and reporting for the past decades for being reliable, simple, and well-suited for many applications. The multicore FBG sensing systems have the ability to respond to a wide variety of measurand, resistance to harsh environments, avoidance of electric sparks, as well as the ease of integration into large-scale fiber networking and communication systems [146], thus making them suitable for a variety of applications, including SHM of dams, highways, bridges, railways, aircraft wings, as well as spacecraft fuel tanks.

### 3.4. Other Examples of SDM Sensors

Last but not least, other prospective cases of SDM sensing systems include the whispering gallery modes for fiber profiling and chemical species measurements, the screw/twisted modes for examining water quality, and the optical beam shaping to improve cantilever deflection measurements.

#### 3.4.1. Whispering Gallery Modes for Chemical Species Measurements

**Figure 20:**
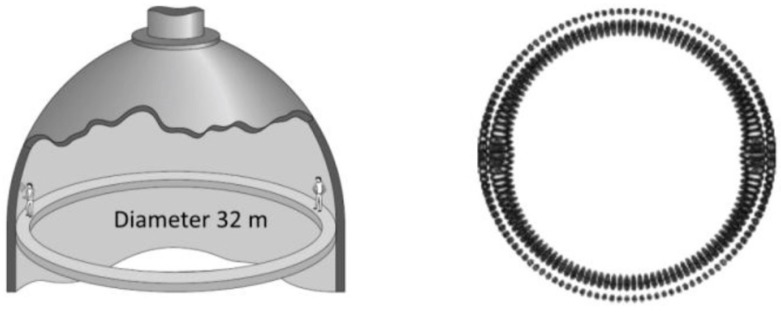
(**Left**) The whispering gallery under a dome of St. Paul’s cathedral and (**Right**) the sound intensity profile showing the whispering gallery mode (WGM) [148].

As mentioned above, the whispering-gallery modes (WGMs) are confined by quasi-total internal reflection along the material interface with virtually cropping incidence patterns, and generated in dielectric microsphere with high-pitched optical resonances in lower refractive index medium [147], while Figure 20 shows the intensity profile of the WGMs [148]. As WGMs with small mode-volume and strong confinement may orbit for many times before escaping the resonator, these modes have been confirmed to provide greatly enhanced detection sensitivity with regard to the refractive index variations of the sensing environment compared with the conventional planar surface-based approaches, with enhanced spontaneous emission threshold-less lasing [149]. Besides other applications in telecommunications, photonics and quantum electrodynamics, such as high-efficiency optical frequency combs, WGMs have also been applied in various sensing applications, including temperature, pressure and force sensors, etc. [150]. In particular, recently WGMs have attracted considerable attention due to their applications in species concentration and biochemical sensing by exploiting sharp photonic resonances, including label-free detection of macromolecules such as proteins and DNA, as well as bacteria and animal cells with accurate permittivity and dielectric loss [9,151]. Such SDM sensors are realized using tapered fibers or prism couplers via coating a zeolite film on the external surface of an optical microsphere with target biomolecules attached on the sphere surface, attributable to the sensitivity of their evanescent field to the refractive index changes of nearby entities [152,153,154].

#### 3.4.2. Screw/Twisted Modes for Examining Water Quality

The screwed or twisted modes, i.e., the OAM modes, are defined as a phase structure in light beams with a local skew angle of the Poynting vector, which can be converted thru nonlinear processes such as second harmonic generation (SHG) or parametric down-conversion (PDC) [155,156]. Such optical vortex with helical phase-fronts can be observed using interference fringes, which can be applied for the translational motion detection of various surfaces and fluids [157]. Specifically, the screwed modes can be used for examining water quality thru the laser spectroscopic approaches in the bulk regions or at the heterogeneous interface of liquid water droplets [158]. When a highly energetic laser pulse is shooting at the target samples as the excitation source to produce the absorption spectrum, the dielectric micro-particles would be rotated and trapped due to the scattering based on the intermolecular interaction between the OH radical and water molecule, whereas the OAM modes would be partially quenched due to the corresponding water asymmetric stretch and OH radical stretch, depending on the quality and purity of water sample [159]. Likewise, such measurement method works on the gaseous environment of the atmosphere or ice crystals [160]. The benefits of using screwed modes in the laser spectroscopic sensing systems compared with the conventional approach have been summarized in Table 3 below.

#### 3.4.3. Optical Beam Shaping for Improving Cantilever Deflection Measurements

**Figure 21:**
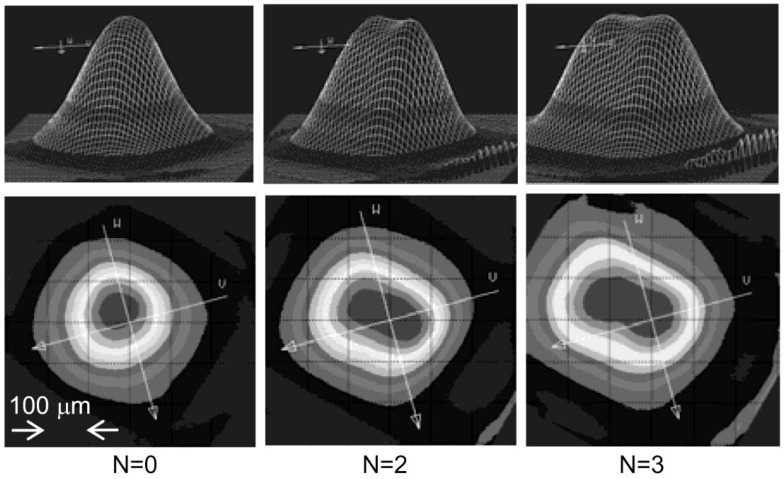
Examples of the observed beam profile reflected at a spatial light phase modulator (SLPM) [161].

Last of all, the optical beam profiles can be modified easily by a spatial light phase modulator (SLPM), while the examples of the observed beam profile reflected at the SLPM are presented in Figure 21, with N as the number of the rotated micro-mirror in series [161]. The micro-cantilevers in atomic force microscopes (AFM) could be employed as ultrasensitive sensors to measure biochemical reactions via surface stress imaging as well as temperature fluctuations [162,163]. Such detection system can be tailored thru optical beam shaping techniques to further boost the accuracy of cantilever deflection measurements, while the relationship between the cantilever deflection and the photo-sensitive detector (PSD) measurement can be simply linearized by means of geometric optics arrangement and standard vector analysis of the optical beam/cantilever [10,164].

## 4. Prospective Outlook

In this section, a prospective outlook for the summary, challenges and further opportunities of SDM optical sensing technologies has been provided, including various markets and applications for the SDM technologies, multiplexing merits in sensing system designs, component cost comparison for SDM measurement systems, as well as the effects of noise and nonlinearity upon the overall performance.

### 4.1. Summary and Comparison

In this subsection, various markets and applications of SDM-based sensing systems are explored first. On the other hand, how these complex mode multiplexing techniques can improve the already working fiber-optic sensor techniques is discussed as well.

#### 4.1.1. SDM Sensing Systems for Various Markets and Applications

The wide range of applications for SDM-based measurement systems are covered in this subsection. The distributed optical sensors using FMFs are quite useful in civil and geotechnical structure health monitory, safety for tunnels, bridges, dams, pipelines, dikes and buildings, fire detection, well-integrity monitoring as well as downhole seismic acquisition. The core multiplexing based systems are popular in the fields of oil and gas production, power cable monitoring, leakage detection at dikes and dams, integrity of liquid natural gas (LNG) carriers and terminals, railway safety monitoring. FBG sensors based on multiplexing are suitable for structure health monitoring of dams, highways, bridges, railways, aircraft wings, spacecraft fuel tanks, and pressure, displacement, acceleration monitoring. Whispering gallery modes are particularly advantageous for label-free detection of macromolecules such as proteins and DNA, as well as bacteria and animal cells, while the screw or twisted modes are for examining water quality, gaseous environment of the atmosphere, ice crystals, as well as motion detection of various surfaces and fluids. Last but not least, optical beam shaping can be used for measuring biochemical reactions through surface stress imaging, and improving cantilever deflection measurements of atomic force microscopes (AFM). A comparison table covering the examples of SDM sensors, their measured parameters, as well as the corresponding sensor applications is shown in Table 4.

#### 4.1.2. Multiplexing Merits in Sensing System Designs

The subsection concentrates on explaining how these complex mode/core multiplexing techniques could improve the existing fiber-optic sensor techniques such as distributed temperature sensing (DTS) and distributed acoustic sensing (DAS). As mentioned earlier, in conventional SMF-based sensing systems, DTS is dedicated to determine only the local temperature based on Raman scattering, while DAS typically provides strain determinations via Rayleigh scattering [41]. For SDM-based systems using FMF or MCF, each of the modes or cores within the sensing medium can serve as an orthogonal interrogator or geophone for one specific sensing parameter, thus responding to an extensive variety of measurands simultaneously. For example, as shown in Table 5 below, since MMF has a higher backscattering coefficient than SMF, the DTS systems with multiplexing can avoid the usage of high-peak power pulses for input, while providing enhanced spatial resolution [142]. Meanwhile, due to modal dispersion and nonlinearity accumulations, the DTS systems with multiplexing are more intended for short-to-medium sensing distance, while SMF-based systems are more suitable for long/ultra-long distance. As for DAS, since Rayleigh scattering depends on a random collection of phases, mode coupling and amplified spontaneous emission (ASE) noise would be added to each channel, making it a bit difficult to improve sensitivity. However, this issue can be easily resolved via code modulation using MIMO DSP or precoding schemes [35,116].

Moreover, for different types of mode multiplexing, including LP modes, supermodes, principle modes, transverse modes, screw/twisted modes, whispering gallery modes, as well as the modes of capillary fibers, their sensing parameters, mode conversion techniques and operation mechanism are summarized in Table 6, while their corresponding benefits and key components are described in Table 7 below.

### 4.2. Challenges for SDM Measurement Systems

This subsection focuses on the possible challenges for SDM-based sensing systems, including the cost efficiency issue, as well as the impact of loss and nonlinearity on system performance.

#### 4.2.1. Component Cost for SDM Sensing Systems

The cost efficiency issue is probably the key element that will lead to or not to the use of SDM techniques in practical fiber-optic sensing systems, which is highly dependent on the development of cost-effective components like light sources and detection units. Technically, lasers with a narrow linewidth (high degree of mono-chromaticity) are desired for fiber-optic sensors as light sources to ensure better resolution. The comparison of wavelength region, output power, linewidth and average cost for different types of lasers in SDM-based measurement systems is summarized in Table 8, from which the laser linewidth from stabilized advanced lasers can be very narrow and reach down to even less than 1 kHz.

Moreover, the component cost scale comparison for SDM sensing systems, including light sources, mode converters/multiplexers, multicore elements, amplifiers and detection units, has been presented in Table 9 above, with each star symbol representing roughly $1,000.00–$3,000.00 depending on the specific applications. The development of SDM-based sensing systems is pushing the boundaries of high-speed multi-wavelength opto-electronic devices and modules, making low-cost optical components possible for SDM implementation. For instance, the opto-electronic sources can be integrated on the silicon platform. Thus the cost of commercialized SDM-based measurement systems is expected to become more compatible with that of standard approaches using SMF and PCF in a broad range of applications in the near future.

#### 4.2.2. The Effects of Noise and Nonlinearity on SDM Sensing Systems

**Figure 22:**
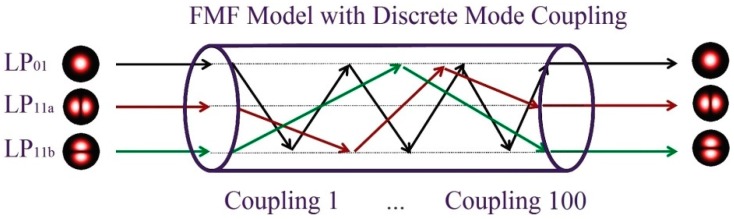
Schematic of spatial modes propagating in a FMF.

In this subsection, some potential performance limitations of SDM-based fiber-optic sensors are further discussed. Since this is a nascent field of research, there is still much unexplored area, involving the effects of noise and nonlinearity. One of the main concerns could be the mode coupling effects, either induced by the index perturbation along the fiber (between non-degenerate modes), or due to the deviations on the transverse index profile (between degenerate modes) [165]. The most common coupling between non-degenerate modes in a FMF is schematically plotted in Figure 22, whereas multiple parallel straight lines symbolize the non-interacting trajectories of two spatial modes. As mode coupling is due to random longitudinal index fluctuation induced by manufacturing process and micro-bending in the cable, the coupling location and strength are random distributed along the fiber [166]. Mode coupling usually leads to mode group delay (MGD) and crosstalk between mode channels, which might degrade the performance of the SDM systems [167]. Other major noise or nonlinearity might include ASE, self-phase modulation, as well as intermodal four-wave mixing, whereas further studies are warranted to resolve these effects [168].

## 5. Concluding Remarks

This paper presents a comprehensive and systematic overview of spatial-division multiplexing (SDM) based fiber-optic sensors concerning a number of aspects in terms of operation principle, fabrication methods, experimental design, and sensing applications. The examples of SDM-based sensing systems include mode-division multiplexing (MDM) using few-mode fiber (FMF), core multiplexing using multicore fiber (MCF) or fiber Bragg grating (FBG), whispering gallery modes for fiber profiling and chemical species measurements, the twisted modes for examining water quality, as well as optical beam shaping to enhance cantilever deflection measurements. Since this is a nascent field of research, there might still be much unexplored area, involving the effects of noise and nonlinearity. As for the cost efficiency issue, which is probably the key element that will lead to or not to the use of SDM in real fiber-optic sensing systems, such systems have the potential to significantly reduce the cost and complexity of parallel systems, which is dependent on the development of highly-integrated and cost-effective components just round the corner. Thus the cost of commercialized SDM-based measurement systems is expected to become more compatible with that of standard approaches using single-mode fibers (SMF) and photonic crystal fibers (PCF), in a broad range of applications including temperature, refractive index, pressure, acoustic/seismic waves and strain sensing in the near future.

## Figures and Tables

**Table 1:** Comparison of *f*-T and *f*-ε coefficients in FMF.

Mode	*C_T_* (MHz/°C)	*C_ε_* (kHz/*με*)
LP_01_	1.29	58.5
LP_11_	1.25	57.6

**Table 2:** Measured value *λ*_1_, *λ*_2_, determined temperature, and strain using MCF [139].

$\lambda_{1}\;\left(nm\right)$	$\lambda_{2}\;\left(nm\right)$	T (°C)	S (*με*)
1528.244	1524.864	33.5	1210.5
1527.560	1524.356	34.3	1868.4

**Table 3:** The benefits of using screwed modes in the laser spectroscopic sensing systems in comparison with the conventional approach.

	Conventional Laser Spectroscopic Approach	Advanced Laser Spectroscopic Sensing System Using Screwed Modes
**Pros**	Non-intrusive remote sensing Monitor concentration in gas phase Compact, robust and affordable in harsh operating environments	❖ Increases the overall number of parallel channels, each as an individual sensor ❖ Appropriate for the detection of broadband multiple absorption lines ❖ Higher sensitivity and selectivity ❖ Better spectral efficiency and reach ❖ More modes → error correction → noise reduction ❖ Improves signal contrast by Encoding, such as code modulation
**Cons**	Only one spatial component of the field vectors captured Relies on small change in power Trade-off between sensitivity and selectivity due to limited wavelength/mode(s) Sensitivity deteriorated by noise	❖ Needs intensive signal processing ❖ Complexity of OAM measurement (That’s why we need MIMO DSP) ❖ Lack of theory for OAM features in specific laser spectroscopic system.

**Table 4:** SDM sensing systems for various markets and applications.

Example of SDM-Based Sensing Systems	Corresponding Markets and Applications	Measured Parameters	Reference
Distributed sensors based on mode-division multiplexing	Civil and geotechnical structure health monitory, safety for tunnels, bridges, dams, pipelines, dikes and buildings, fire detection, well-integrity monitoring and downhole seismic acquisition	Temperature; Strain; Pressure; Stress; Force; Acoustic; Vibration; Bending; Refractive index	[14,28,38,127]
Distributed sensors based on core multiplexing	Oil and gas production, power cable monitoring, leakage detection at dikes and dams, integrity of liquid natural gas (LNG) carriers and terminals, railway safety monitoring	Temperature; Strain; Pressure; Stress; Vibration; Bending; Shape; Displacement	[49,80,136,137,138,139,140]
Fiber Bragg grating sensors based on multiplexing	Structure health monitoring of dams, highways, bridges, railways, aircraft wings, as well as spacecraft fuel tanks; pressure, displacement, or acceleration monitoring	Temperature; Strain; Pressure; Bending; Shape; Displacement; Acceleration	[84,141,142,143,144,145]
Whispering gallery modes for chemical species measurements	Label-free detection of macromolecules such as proteins and DNA, as well as bacteria and animal cells; temperature, pressure sensors	Temperature, Pressure, Force, Refractive Index, Species Concentration, Biochemical Compounds	[9,147,148,149,150,151,152]
Screw/twisted modes for examining water quality	Examining water quality, gaseous environment of the atmosphere, ice crystals; atmospheric turbulence monitoring, motion detection of various surfaces and fluids.	Species Concentration, Biochemical Compounds, Shape; Displacement; Acceleration	[53,54,55,70,71,155,156,157,158,159]
Optical beam shaping for improving cantilever deflection measurements	Cantilever deflection measurements, atomic force microscopes (AFM), measuring biochemical reactions via surface stress imaging and temperature fluctuations	Temperature; Species Concentration, Biochemical Compounds; Refractive Index	[10,162,163,164]

**Table 5:** Multiplexing comparison in DTS and DAS sensing system designs.

	DTS	DAS
With Multiplexing	❖ Have higher backscattering coefficients ❖ Avoids the usage of high peak power pulses for input ❖ Provides better spatial resolution ❖ Modal dispersion slightly degrade spatial resolution mainly for short-to-medium distance	❖ Mode coupling and ASE noise in each mode added to the system ❖ Difficult to align all modes correctly ❖ Challenging to improve sensitivity ❖ Signals in different modes propagate in different speeds, only for short-to-medium distance
Without Multiplexing	❖ Have lower backscattering coefficients ❖ Requires high peak power pulses ❖ Provides worse spatial resolution ❖ More suitable for long/ultra-long distance due to lack of modal dispersion accumulation	❖ No mode coupling, Need to reduce ASE noise in fundamental mode ❖ Obtain trace by launching a single pulse ❖ May provide higher spatial resolution ❖ More suitable for long distance due to lack of modal dispersion accumulation

**Table 6:** Comparison of sensing parameters, mode conversion techniques and operation mechanism for different types of mode multiplexing.

Corresponding Modes	Sensing Parameters	Mode Conversion Techniques	Sensing Mechanism
LP modes	Temperature; Strain; Pressure; Acoustic; Vibration; Bending; Refractive index; Humidity	Phase plates; FBG; LCoS; fused fiber coupler; FWM	Brillouin/Raman/Rayleigh scattering or spectral shift from FBG
Supermodes	Temperature; Strain; Acoustic; Curvature; Bending; Refractive index	Phase plates; FBG; LCoS	Using either mode/core correlation or spectral shift for sensor interrogation
Principle modes	Temperature; Strain; Bending	Phase plates; LPG; Spatial light modulator	Spatial modes without modal dispersion to first-order in frequency
Transverse modes	Temperature; Strain; Pressure; Acoustic; Bending;	Phase plates; LPG; LCoS;	Brillouin/Raman/Rayleigh scattering or spectral shift from FBG
Screw/twisted modes	Atmospheric turbulence monitoring; lateral motion detecting; biomedical imaging	Cylindrical lenses; Helical gratings; parametric oscillator	OAM states partially quenched due to inter-molecular interaction
Whispering gallery modes	Temperature, refractive index, biochemical species	Whispering gallery mode resonator in a tapered fiber	Travel around concave surfaces with low loss due to quantum tunneling
Modes of capillary optical fibers	Temperature; Strain; Flow rate, pulling force, fiber geometry, biochemical species	Capillary tapered mode converter	Multiple modes excited/interfered to form fringes collected by lead-out SMF

**Table 7:** Comparison of key components and multiplexing benefits using different types of modes.

Corresponding Modes	Measurement Components	Benefits	References
LP modes	Using FMF, MMF, MCF itself as the sensing medium with direct/coherent detection	Simple; Compact; low loss; high sensitivity; good repeatability	[14,24,64,77,122]
Supermodes	A few-millimeter-long piece of seven-core fiber spliced between two single-mode fibers	Compact; low loss; high sensitivity; good repeatability	[49,67,69]
Principle modes	A multimode waveguide system in the vicinity of the phase-matching frequency	High speed; high sensitivity; low modal dispersion	[48,50,51]
Transverse modes	Using FMF, MMF, MCF itself as the sensing medium with direct/coherent detection	Compact; low loss; high sensitivity; good repeatability	[28,52,130]
Screw/twisted modes	Laser spectroscopic devices; atomic force microscopes; photo-sensitive detector	Higher sensitivity and selectivity; Better spectral efficiency	[53,54,55,70,71,153,154,155,156,157]
Whispering gallery modes	A microscopic glass sphere from micro-cavities of optical fiber resonator	High sensitivity to refractive index; useful in biochemical sensing	[56,57,72,73,147,148,149,150,151,152]
Modes of capillary optical fibers	A fused-silica capillary and FBG sandwiched by single-mode fibers	High sensitivity to refractive index; useful in biochemical sensing	[58,59]

**Table 8:** Comparison of linewidth and cost for different types of lasers in SDM-based systems.

Type of Light Sources	Wavelength Region	Output Power	Linewidth	Cost
Distributed feedback laser diodes (DFB lasers)	1000 nm–1500 nm	tens of mW	several MHz	$300.00–$3,500.00
Distributed Bragg reflector lasers (DBR lasers)	1000 nm–1500 nm	tens of mW	several MHz	$500.00–$3,950.00
Fabry-Perot Laser Diodes (FP lasers)	400 nm–1550 nm	10–300 mW	1–2 MHz	$1,475.00–$4,000.00
distributed feedback fiber lasers (DFB + FBG)	980 nm–1550 nm	20–150 mW	a few kHz	About $5,000.00
InGaAsP/InP distributed feedback laser	1064 nm–1560 nm	25 mW–300 mW	10 kHz	About $6,000.00
Nd:YAG laser	1064 nm–1550 nm	100 mW–3 W	10 kHz	About $10,000.00
Diode-pumped solid-state bulk lasers	1064 nm–1550 nm	100 mW–1 W	a few kHz	About $14,900.00
Distributed Feedback Quantum Cascade Lasers (QCLs)	760 nm–1600 nm	100 mW–5 W	a few hundred Hz	$6,200.00–$15,000.00

**Table 9:** Comparison regarding proposed sensor component costs for SDM sensing systems.

Component Type	Components	Cost
	Distributed feedback laser diodes (DFB lasers)	★
Light source	Fabry-Perot laser diodes (FP lasers)	★★
	Quantum cascade lasers (QCLs)	★★★
	Nd:YAG lasers	★★★★
	Long-period grating (LPG) based converter	★
	Liquid crystal on silicon (LCoS) panels	★★
	Thin phase plates	★★
Mode converter	Helical gratings (HGs)	★★
	Cylindrical lenses	★★★
	Optical parametric oscillator	★★★
	Whispering gallery mode resonator	★★★★
	Capillary tapered mode converter	★★★★
Multiplexer	Spatial light modulators (SLM) via LCoS	★★
	Photonic lantern (PL)	★★★
	LPG based multicore elements	★★
Multicore elements	Asymmetrical coupler based multicore elements	★★★
	Special fiber based multicore elements	★★★★
	Few-mode Raman amplifiers	★★
SDM amplifiers	Few-mode erbium-doped fiber amplifiers (FM-EDFA)	★★★
	Multi-core EDFAs	★★★★
	Direct detection	★★
Detection units	Homodyne detection	★★★★
	Heterodyne detection	★★★★

★ representing roughly $1,000.00–$3,000.00 depending on the specific applications.

## References

[1] Winzer P.J. (2014). Making spatial multiplexing a reality. Nat. Photonics.

[2] Essiambre R.-J., Kramer G., Winzer P.J., Foschini G.J., Goebel B. (2010). Capacity limits of optical fiber networks. J. Lightwave Technol..

[3] Richardson D.J., Fini J.M., Nelson L.E. (2013). Space-division multiplexing in optical fibres. Nat. Photonics.

[4] Yaman F., Bai N., Zhu B., Wang T., Li G. (2010). Long distance transmission in few-mode fibers. Opt. Express.

[5] Van Uden R.G.H., Correa R.A., Lopez E.A., Huijskens F.M., Xia C., Li G., Schülzgen A., Waardt H.D., Koonen A.M.J., Okonkwo C.M. (2014). Ultra-high-density spatial division multiplexing with a few-mode multicore fibre. Nat. Photonics.

[6] Pan Z., He X., Weng Y. Hardware efficient frequency domain equalization in few-mode fiber coherent transmission systems. Proceedings of the SPIE; Next-Generation Optical Communication: Components, Sub-Systems, and Systems III.

[7] Grattan K.T.V., Sun T. (2000). Fiber optic sensor technology: An overview. Sens. Actuators A Phys..

[8] Murshid S., Grossman B., Narakorn P. (2008). Spatial domain multiplexing: A new dimension in fiber optic multiplexing. Opt. Laser Technol..

[9] Boleininger A., Lake T., Hami S., Vallance C. (2010). Whispering gallery modes in standard optical fibres for fibre profiling measurements and sensing of unlabelled chemical species. Sensors.

[10] Beaulieua L.Y., Godin M., Larochec O., Tabard-Cossac V., Grutter P.A. (2007). Complete analysis of the laser beam deflection systems used in cantilever-based systems. Ultramicroscopy.

[11] Borecki M., Korwin-Pawlowski M.L., Beblowska M., Szmidt J., Szmidt M., Duk M., Urbańska K., Jakubowski A., Einschlag F.S.G. (2011). Intelligent photonic sensors for application, in decentralized wastewater systems. Waste Water—Evaluation and Management.

[12] Weng Y., He X., Wang J., Pan Z. All-optical ultrafast wavelength and mode converter based on inter-modal nonlinear wave mixing in few-mode fibers. Proceedings of the Conference on Lasers and Electro-Optics (CLEO).

[13] Carboni C., Li G. (2016). Novel applications of space-division multiplexing. Front. Optoelectron..

[14] Li A., Wang Y., Fang J., Li M.-J., Kim B.Y., Shieh W. (2015). Few-mode fiber multi-parameter sensor with distributed temperature and strain discrimination. Opt. Lett..

[15] Tang M., Zhao Z., Gan L., Wu H., Wang R., Li B., Fu S., Liu H., Liu D., Wei H. Spatial-division multiplexed optical sensing using MCF and FMF. Proceedings of the Advanced Photonics Congress 2016.

[16] Gloge D. (1971). Weakly guiding fibers. Appl. Opt..

[17] Richardson D.J. (2010). Filling the light pipe. Science.

[18] Grattan L.S., Meggitt B.T. (1998). Optical Fiber Sensor Technology: Devices and Technology.

[19] Antonelli C., Mecozzi A., Shtaif M., Winzer P.J. (2012). Stokes-space analysis of modal dispersion in fibers with multiple mode transmission. Opt. Express.

[20] Agrawal G.P. (2010). Fiber-Optic Communication Systems.

[21] Pan Z., Weng Y., He X., Wang J. Adaptive frequency-domain equalization and MIMO signal processing in mode division multiplexing systems using few-mode fibers. Proceedings of the Signal Processing in Photonic Communications (SPPCom).

[22] Rao Y.J., Ran Z.L., Zhou C.X. (2006). Fiber-optic Fabry-Perot sensors based on a combination of spatial-frequency division multiplexing and wavelength division multiplexing formed by chirped fiber Bragg grating pairs. Appl. Opt..

[23] Berdagué S., Facq P. (1982). Mode division multiplexing in optical fibers. Appl. Opt..

[24] Weng Y., Ip E., Pan Z., Wang T. Few-mode distributed optical fiber sensors. Proceedings of the Advanced Photonics Congress 2015.

[25] Carpenter J., Thomsen B.C., Wilkinson T.D. (2012). Degenerate mode-group division multiplexing. J. Lightwave Technol..

[26] Chen D., Wu C., Tse M.L.V., Tam H.-Y. (2011). Hydrostatic pressure sensor based on mode interference of a few mode fiber. Prog. Electromagn. Res..

[27] Sun A., Wu Z. (2015). Multimode interference in single mode–multimode FBG for simultaneous measurement of strain and bending. IEEE Sens. J..

[28] Li A., Wang Y., Hu Q., Che D., Chen X., Shieh W. (2014). Measurement of distributed mode coupling in a few-mode fiber using a reconfigurable Brillouin OTDR. Opt. Lett..

[29] Pan Z., Weng Y., Wang J. (2016). Investigation of nonlinear effects in few-mode fibers. Photonic Netw. Commun..

[30] Bao X., Chen L. (2012). Recent progress in distributed fiber optic sensors. Sensors.

[31] Spillman W.B. (1982). Multimode fiber-optic pressure sensor based on the photoelastic effect. Opt. Lett..

[32] Parker T.R., Farhadiroushan M., Handerek V.A., Rogers A.J. (1997). Temperature and strain dependence of the power level and frequency of spontaneous Brillouin scattering in optical fibers. Opt. Lett..

[33] Alahbabi M.N., Cho Y.T., Newson T.P. (2005). Simultaneous temperature and strain measurement with combined spontaneous Raman and Brillouin scattering. Opt. Lett..

[34] Liu X., Bao X. (2012). Brillouin spectrum in LEAF and simultaneous temperature and strain measurement. J. Lightwave Technol..

[35] Wang L., LaRochelle S. (2015). Design of eight-mode polarization-maintaining few-mode fiber for multiple-input multiple-output-free spatial division multiplexing. Opt. Lett..

[36] Souza K.D. (2006). Significance of coherent Rayleigh noise in fibre-optic distributed temperature sensing based on spontaneous Brillouin scattering. Meas. Sci. Technol..

[37] Wood T.H., Linke R.A., Kasper B.L., Carr E.C. (1988). Observation of coherent Rayleigh noise in single-source bidirectional optical fiber systems. J. Lightwave Technol..

[38] Song K.Y., Kim Y.H. (2013). Characterization of stimulated Brillouin scattering in a few-mode fiber. Opt. Lett..

[39] Tanimola F., Hill D. (2009). Distributed fibre optic sensors for pipeline protection. J. Nat. Gas Sci. Eng..

[40] Lopez-Higuera J.M., Rodriguez Cobo L., Incera A.Q., Cobo A. (2011). Fiber optic sensors in structural health monitoring. J. Lightwave Technol..

[41] Bolognini G., Hartog A. (2013). Raman-based fibre sensors: Trends and applications. Opt. Fiber Technol..

[42] Golub M.A., Shwartz S., Ruschin S. Space-division multiplexing of coherent beams by diffractive optical elements. Proceedings of the Optical Fiber Communication (OFC).

[43] Demas J., Rishøj L., Ramachandran S. (2015). Free-space beam shaping for precise control and conversion of modes in optical fiber. Opt. Express.

[44] Shwartz S., Golub M., Ruschin S. (2013). Diffractive optical elements for mode-division multiplexing of temporal signals with the aid of Laguerre–Gaussian modes. Appl. Opt..

[45] Mayeh M., Farahi F. (2011). Laser beam shaping and mode conversion in optical fibers. Photonic Sens..

[46] Weng Y., He X., Wang J., Pan Z. (2015). All-optical ultrafast wavelength and mode converter based on inter-modal four-wave mixing in few-mode fibers. Opt. Commun..

[47] Chen H., Uden R.V., Okonkwo C., Koonen T. (2014). Compact spatial multiplexers for mode division multiplexing. Opt. Express.

[48] Fan S., Kahn J.M. (2005). Principal modes in multimode waveguides. Opt. Lett..

[49] Ziolowicz A., Bienkowska B., Budnicki D., Jozwik M., Ostrowski L., Murawski M., Pytel A., Tenderenda T., Wojcik G., Szostkiewicz L. Supermode interference in dual-core hole-assisted fiber for sensing. Proceedings of the SPIE Optical Fibers and Their Applications 2015.

[50] Carpenter J., Eggleton B.J., Schröder J. (2015). Observation of Eisenbud–Wigner–Smith states as principal modes in multimode fibre. Nat. Photonics.

[51] Milione G., Nolan D.A., Alfano R.R. (2015). Determining principal modes in a multimode optical fiber using the mode dependent signal delay method. J. Opt. Soc. Am. B.

[52] Tucker J.R., Rakić A.D., O’Brien C.J., Zvyagin A.V. (2007). Effect of multiple transverse modes in self-mixing sensors based on vertical-cavity surface-emitting lasers. Appl. Opt..

[53] Chen C., Yang H., Tong S., Lou Y. (2016). Changes in orbital-angular-momentum modes of a propagated vortex Gaussian beam through weak-to-strong atmospheric turbulence. Opt. Express.

[54] Cvijetic N., Milione G., Ip E., Wang T. (2015). Detecting lateral motion using light’s orbital angular momentum. Sci. Rep..

[55] Weng Y., Pan Z. Orbital-angular-momentum-based image sensor using high resolution photoacoustic tomography. Proceedings of the Advanced Photonics.

[56] Matsko A.B., Ilchenko V.S. (2006). Optical resonators with whispering gallery modes I: Basics. IEEE JSTQE.

[57] Foreman M.R., Swaim J.D., Vollmer F. (2015). Whispering gallery mode sensors. Adv. Opt. Photonics.

[58] Sotsky A.B., Sotskaya L.I. (2004). Modes of capillary optical fibers. Opt. Commun..

[59] Dutt A., Mahapatra S., Varshney S.K. (2011). Capillary optical fibers: Design and applications for attaining a large effective mode area. J. Opt. Soc. Am. B.

[60] Flamm D., Naidoo D., Schulze C., Forbes A., Duparré M. (2012). Mode analysis with a spatial light modulator as a correlation filter. Opt. Lett..

[61] Labroille G., Denolle B., Jian P., Genevaux P., Treps N., Morizur J.-F. (2014). Efficient and mode selective spatial mode multiplexer based on multi-plane light conversion. Opt. Express.

[62] Hoyningen-Huene J.V., Ryf R., Winzer P. (2013). LCoS-based mode shaper for few-mode fiber. Opt. Express.

[63] Salsi M., Koebele C., Sperti D., Tran P., Mardoyan H., Brindel P., Bigo S., Boutin A., Verluise F., Sillard P. (2012). Mode-division multiplexing of 2 × 100 Gb/s channels using an LCOS-based spatial modulator. J. Lightwave Technol..

[64] Li A., Chen X., Amin A.A., Shieh W. (2012). Fused fiber mode couplers for few-mode transmission. IEEE Photonics Technol. Lett..

[65] Weng Y., He X., Wang J., Zhu B., Pan Z. Mode and Wavelength Conversion Based on Inter-Modal Four-Wave Mixing in a Highly Nonlinear Few-Mode Fiber. Proceedings of the Signal Processing in Photonic Communications (SPPCOM).

[66] Li G. (2009). Recent advances in coherent optical communication. Adv. Opt. Photonics.

[67] Peral E., Yariv A. (2002). Supermodes of grating-coupled multimode waveguides and application to mode conversion between copropagating modes mediated by backward Bragg scattering. J. Lightwave Technol..

[68] Giles I., Obeysekara A., Chen R., Giles D., Poletti F., Richardson D. (2012). Fiber LPG mode converters and mode selection technique for multimode SDM. IEEE Photonics Technol. Lett..

[69] Xia C., Bai N., Ozdur I., Zhou X., Li G. (2011). Supermodes for optical transmission. Opt. Express.

[70] Fang L., Wang J. (2016). Mode Conversion and Orbital Angular Momentum Transfer among Multiple Modes by Helical Gratings. IEEE J. Quantum Electron..

[71] Martinelli M., Huguenin J.A.O., Nussenzveig P., Khoury A.Z. (2004). Orbital angular momentum exchange in an optical parametric oscillator. Phys. Rev. A.

[72] Huang L., Wang J., Peng W., Zhang W., Bo F., Yu X., Gao F., Chang P., Song X., Zhang G. (2016). Mode conversion in a tapered fiber via a whispering gallery mode resonator and its application as add/drop filter. Opt. Lett..

[73] Farnesi D., Barucci A., Righini G.C., Berneschi S., Soria S., Nunzi Conti G. (2014). Optical frequency conversion in silica-whispering-gallery-mode micro-spherical resonators. Phys. Rev. Lett..

[74] Saitoh F., Saitoh K., Koshiba M. (2010). A design method of a fiber-based mode multi/demultiplexer for mode-division multiplexing. Opt. Express.

[75] Bouchal Z., Haderka O., Celechovsky R. (2005). Selective excitation of vortex fiber modes using a spatial light modulator. New J. Phys..

[76] Li G., Bai N., Zhao N., Xia C. (2014). Space-division multiplexing: The next frontier in optical communication. Adv. Opt. Photonics.

[77] Li A., Hu Q., Che D., Wang Y., Shieh W. Measurement of distributed mode coupling in a few-mode fiber using a Brillouin optical time domain reflectometer. Proceedings of the European Conference on Optical Communication (ECOC).

[78] Fontaine N.K., Ryf R., Bland-Hawthorn J., Leon-Saval S.G. (2012). Geometric requirements for photonic lanterns in space division multiplexing. Opt. Express.

[79] Leon-Saval S.G., Fontaine N.K., Salazar-Gil J.R., Ercan B., Ryf R., Bland-Hawthorn J. (2014). Mode-selective photonic lanterns for space-division multiplexing. Opt. Express.

[80] Napierala M., Murawski M., Szymanski M., Ostrowski L., Szostkiewicz L., Mergo P., Jaroszewicz L., Nasilowski T. Optical fiber elements for addressing individual cores in multicore optical fiber sensors. Proceedings of the 23rd International Conference on Optical Fibre Sensors.

[81] Korotky S.K. (2012). Price-points for components of multi-core fiber communication systems in backbone optical networks. J. Opt. Commun. Netw..

[82] Jain S., Rancaño V.J.F., May-Smith T.C., Petropoulos P., Sahu J.K., Richardson D.J. (2014). Multi-element fiber technology for space-division multiplexing applications. Opt. Express.

[83] Uchiyama T., Hamada N., Cai C. (2014). Highly sensitive CMOS magnetoimpedance sensor using miniature multi-core head based on amorphous wire. IEEE Trans. Magn..

[84] Saffari P., Allsop T., Adebayo A., Webb D., Haynes R., Roth M.M. (2014). Long period grating in multicore optical fiber: An ultra-sensitive vector bending sensor for low curvatures. Opt. Lett..

[85] Chen M.-Y., Wei J., Sheng Y., Ren N.-F. (2016). Design and optimization of fundamental mode filters based on long-period fiber gratings. Opt. Fiber Technol..

[86] Wolinski T.R., Lesiak P., Domanski A.W. (2008). Polarimetric optical fiber sensors of a new generation for industrial applications. Bull. Pol. Acad. Sci. Tech. Sci..

[87] Yuan L., Wang X. (2007). Four-beam single fiber optic interferometer and its sensing characteristics. Sens. Actuators A Phys..

[88] Borecki M. (2007). Intelligent fiber optic sensor for estimating the concentration of a mixture-design and working principle. Sensors.

[89] Lane S., West P., François A., Meldrum A. (2015). Protein biosensing with fluorescent microcapillaries. Opt. Express.

[90] Madore W.-J., de Montigny E., Ouellette O., Lemire-Renaud S., Leduc M., Daxhelet X., Godbout N., Boudoux C. (2013). Asymmetric double-clad fiber couplers for endoscopy. Opt. Lett..

[91] Weng Y., Pan Z. An efficient scheme of intermodal distributed Raman amplification using tailored doping profiles in spatial-division multiplexed coherent fiber-optic transmission systems. Proceedings of the SPIE Optical Components and Materials XIII.

[92] Jung Y., Lim E.L., Kang Q., May-Smith T.C., Wong N.H.L., Standish R., Poletti F., Sahu J.K., Alam S.U., Richardson D.J. (2014). Cladding pumped few-mode EDFA for mode division multiplexed transmission. Opt. Express.

[93] Le Cocq G., Bigot L., le Rouge A., Bigot-Astruc M., Sillard P., Koebele C., Salsi M., Quiquempois Y. (2012). Modeling and characterization of a few-mode EDFA supporting four mode groups for mode division multiplexing. Opt. Express.

[94] Bai N., Ip E., Wang T., Li G. (2011). Multimode fiber amplifier with tunable modal gain using a reconfigurable multimode pump. Opt. Express.

[95] Alahbabi M.N., Cho Y.T., Newson T.P. (2005). 150-km-range distributed temperature sensor based on coherent detection of spontaneous Brillouin backscatter and in-line Raman amplification. J. Opt. Soc. Am. B.

[96] Ip E., Li M.-J., Bennett K., Huang Y.-K., Tanaka A., Korolev A., Koreshkov K., Wood W., Mateo E., Hu J. (2014). 146*λ* × 6 × 19-Gbaud Wavelength- and Mode-Division Multiplexed Transmission over 10 × 50-km Spans of Few-Mode Fiber with a Gain-Equalized Few-Mode EDFA. J. Lightwave Technol..

[97] Smith A.V., Smith J.J. (2011). Mode instability in high power fiber amplifiers. Opt. Express.

[98] Antonelli C., Mecozzi A., Shtaif M. (2013). Raman amplification in multimode fibers with random mode coupling. Opt. Lett..

[99] Rottwitt K., Nielsen K., Friis S.M.M., Castaneda M.A.U. Challenges in higher order mode Raman amplifiers. Proceedings of the Optical Fiber Communication (OFC).

[100] Weng Y., Wang T., Pan Z. Optimization of mode-dependent gain efficiency based on intermodal Raman scattering for few-mode distributed Raman amplifier. Proceedings of the Conference on Lasers and Electro-Optics (CLEO).

[101] Abedin K.S., Fini J.M., Thierry T.F., Supradeepa V.R., Zhu B., Yan M.F., Bansal L., Monberg E.M., DiGiovanni D.J. (2014). Multicore erbium doped fiber amplifiers for space division multiplexing systems. J. Lightwave Technol..

[102] Elkin N.N., Napartovich A.P., Troshchieva V.N., Vysotsky D.V. (2007). Mode competition in multi-core fiber amplifier. Opt. Commun..

[103] Abedin K.S., Taunay T.F., Fishteyn M., DiGiovanni D.J., Supradeepa V.R., Fini J.M., Yan M.F., Zhu B., Monberg E.M., Dimarcello F.V. (2012). Cladding-pumped erbium-doped multicore fiber amplifier. Opt. Express.

[104] Liu L., Gong Y., Wu Y., Zhao T., Wu H.-J., Rao Y.-J. (2012). Spatial Frequency Multiplexing of Fiber-Optic Interferometric Refractive Index Sensors Based on Graded-Index Multimode Fibers. Sensors.

[105] Bora M., McCarrick J., Zumstein J., Bond S., Chang A., Moran B., Benett W.J., Bond T. Multiplexed gas spectroscopy using tunable VCSELs. Proceedings of the SPIE Advanced Environmental, Chemical, and Biological Sensing Technologies IX.

[106] Xu J., Hou L., Deng Q., Han L., Liang S., Marsh J.H., Zhu H. (2016). Fully integrated multi-optoelectronic synthesizer for THz pumping source in wireless communications with rich backup redundancy and wide tuning range. Sci. Rep..

[107] Alarcón-Salazar J., Zaldívar-Huerta I.E., Aceves-Mijares M. (2016). An optoelectronic circuit with a light source, an optical waveguide and a sensor all on silicon: Results and analysis of a novel system. Opt. Laser Technol..

[108] Xu F., Wang Y., Li F. (2016). Pixel multiplexing technique for real-time three-dimensional-imaging laser detection and ranging system using four linear-mode avalanche photodiodes. Rev. Sci. Instrum..

[109] Alahbabi M.N., Cho Y.T., Newson T.P. (2004). 100 km distributed temperature sensor based on coherent detection of spontaneous Brillouin backscatter. Meas. Sci. Technol..

[110] He X., Weng Y., Pan Z. (2014). A step-size controlled method for fast convergent adaptive FD-LMS algorithm in few-mode fiber communication systems. J. Lightwave Technol..

[111] Inan B., Spinnler B., Ferreira F., Borne D.V.D., Lobato A., Adhikari S., Sleiffer V.A.J.M., Kuschnerov M., Hanik N., Jansen S.L. (2012). DSP complexity of mode-division multiplexed receivers. Opt. Express.

[112] Okonkwo C., Uden R.V., Chen H., Waardt H.D., Koonen T. (2015). Advanced coding techniques for few mode transmission systems. Opt. Express.

[113] Weng Y., He X., Pan Z. Performance analysis of low-complexity adaptive frequency-domain equalization and MIMO signal processing for compensation of differential mode group delay in mode-division multiplexing communication systems using few-mode fibers. Proceedings of the SPIE; Next-Generation Optical Communication: Components, Sub-Systems, and Systems V.

[114] Luís R.S., Puttnam B.J., Mendinueta J.M.D., Klaus W., Sakaguchi J., Awaji Y., Kawanishi T., Kanno A., Wada N. (2014). OSNR penalty of self-homodyne coherent detection in spatial-division-multiplexing systems. Photonics Technol. Lett..

[115] Randel S., Ryf R., Sierra A., Winzer P.J., Gnauck A.H., Bolle C.A., Essiambre R.-J., Peckham D.W., McCurdy A., Lingle R. (2011). 6 × 56-Gb/s mode-division multiplexed transmission over 33-km few-mode fiber enabled by 6 × 6 MIMO equalization. Opt. Express.

[116] Arik S.Ö., Askarov D., Kahn J.M. MIMO signal processing in mode-division multiplexing systems. Proceedings of the SPIE; Optical Metro Networks and Short-Haul Systems VII.

[117] Weng Y., Wang T., Pan Z. Fast-convergent adaptive frequency-domain recursive least-squares algorithm with reduced complexity for MDM transmission systems using optical few-mode fibers. Proceedings of the Conference on Lasers and Electro-Optics (CLEO).

[118] Kersey A.D., Dandridge A. (1988). Distributed and multiplexed fibre-optic sensor systems. J. Inst. Electron. Radio Eng..

[119] Ryf R., Randel S., Gnauck A.H., Bolle C., Sierra A., Mumtaz S., Esmaeelpour M., Burrows E.C., Essiambre R.-J., Winzer P.J. (2012). Mode-division multiplexing over 96 km of few-mode fiber using coherent 6 × 6 MIMO processing. J. Lightwave Technol..

[120] Taiwo A., Taiwo S., Sahbudin R.K.Z., Yaacob M.H., Mokhtar M. (2014). Fiber vibration sensor multiplexing techniques for quasi-distributed sensing. Opt. Laser Technol..

[121] He X., Weng Y., Wang J., Pan Z. Noise power directed adaptive frequency domain least mean square algorithm with fast convergence for DMGD compensation in few-mode fiber transmission systems. Proceedings of the Optical Fiber Communication (OFC).

[122] Kumar A., Goel N.K., Varshney R.K. (2001). Studies on a few-mode fiber-optic strain sensor based on LP_01_–LP_02_ mode interference. J. Lightwave Technol..

[123] Weng Y., Ip E., Pan Z., Wang T. (2015). Few-mode distributed optical-fiber sensors. Opt. Photonics News.

[124] Ashry I., Wang A., Xu Y. (2016). Mode-division-multiplexing of absorption-based fiber optical sensors. Opt. Express.

[125] Bao X., Chen L. (2011). Recent progress in Brillouin scattering based fiber sensors. Sensors.

[126] Kobyakov A., Sauer M., Chowdhury D. (2010). Stimulated Brillouin scattering in optical fibers. Adv. Opt. Photonics.

[127] Weng Y., Ip E., Pan Z., Wang T. Distributed temperature and strain sensing using spontaneous Brillouin scattering in optical few-mode fibers. Proceedings of the Conference on Lasers and Electro-Optics (CLEO).

[128] Gogolla T., Krebber K. Fiber sensors for distributed temperature and strain measurements using Brillouin scattering and frequency-domain methods. Proceedings of the SPIE Chemical, Biochemical and Environmental Fiber Sensors IX.

[129] Song K.Y., Kim Y.H. Measurement of intramodal and intermodal Brillouin gain spectra in a few-mode fiber. Proceedings of the Optical Fiber Communication (OFC).

[130] Li A., Wang Y., Hu Q., Shieh W. (2015). Few-mode fiber based optical sensors. Opt. Express.

[131] Matsui T., Nakajima K., Yamamoto F. (2015). Guided acoustic-wave Brillouin scattering characteristics of few-mode fiber. Appl. Opt..

[132] Song K.Y., Kim Y.H., Kim B.Y. (2013). Intermodal stimulated Brillouin scattering in two-mode fibers. Opt. Lett..

[133] Weng Y., Ip E., Pan Z., Wang T. (2015). Single-end simultaneous temperature and strain sensing techniques based on Brillouin optical time domain reflectometry in few-mode fibers. Opt. Express.

[134] Mizuno Y., Nakamura K. (2010). Potential of Brillouin scattering in polymer optical fiber for strain-insensitive high-accuracy temperature sensing. Opt. Lett..

[135] Wu H., Wang R., Liu D., Fu S., Zhao C., Wei H., Tong W., Shum P.P., Tang M. (2016). Few-mode fiber based distributed curvature sensor through quasi-single-mode Brillouin frequency shift. Opt. Lett..

[136] Gan L., Wang R., Tang M., Duan L., Li B., Fu S., Tong W., Wei H., Liu D., Shum P.P. Space-division multiplexed multicore fiber Mach-Zehnder interferometer for joint temperature and strain sensing. Proceedings of the Optical Fiber Communication (OFC).

[137] Newkirk A.V., Antonio-Lopez E., Salceda-Delgado G., Piracha M.U., Amezcua-Correa R., Schulzgen A. Simultaneous measurement of strain and temperature using high sensitivity multicore fiber sensors. Proceedings of the Conference on Lasers and Electro-Optics (CLEO).

[138] Newkirk A.V., Antonio-Lopez E., Salceda-Delgado G., Piracha M.U., Amezcua-Correa R., Schulzgen A. (2015). Multicore fiber sensors for simultaneous measurement of force and temperature. Photonics Technol. Lett..

[139] Gan L., Wang R., Liu D., Duan L., Liu S., Fu S., Li B., Feng Z., Wei H., Tong W. (2016). Spatial-division multiplexed Mach–Zehnder interferometers in heterogeneous multicore fiber for multiparameter measurement. IEEE Photonics J..

[140] Mizuno Y., Hayashi N., Tanaka H., Wada Y., Nakamura K. (2015). Brillouin scattering in multicore optical fibers for sensing applications. Sci. Rep..

[141] Fender A., MacPherson W.N., Maier R.R.J., Barton J.S., George D.S., Howden R.I., Smith G.W., Jones B.J.S., McCulloch S., Chen X. (2008). Two-axis temperature-insensitive accelerometer based on multicore fiber Bragg gratings. IEEE Sens. J..

[142] Dochow S., Latka I., Becker M., Spittel R., Kobelke J., Schuster K., Graf A., Brückner S., Unger S., Rothhardt M. (2012). Multicore fiber with integrated fiber Bragg gratings for background-free Raman sensing. Opt. Express.

[143] Lindley E., Min S.-S., Leon-Saval S., Cvetojevic N., Lawrence J., Ellis S., Bland-Hawthorn J. (2014). Demonstration of uniform multicore fiber Bragg gratings. Opt. Express.

[144] Askins C.G., Miller G.A., Friebele E.J. Bend and twist sensing in a multiple-core optical fiber. Proceedings of the Optical Fiber Communication (OFC).

[145] Askins C.G., Miller G.A., Friebele E.J. Bend and twist sensing in a multi-core optical fiber. Proceedings of the 21st Annual Meeting of the IEEE Lasers and Electro-Optics Society (LEOS 2008).

[146] Hotate K., Kajiwara K. (2008). Proposal and experimental verification of Bragg wavelength distribution measurement within a long-length FBG by synthesis of optical coherence function. Opt. Express.

[147] Cros D., Guillon P. (1990). Whispering gallery dielectric resonator modes for W-band devices. IEEE Trans. Microw. Theory Tech..

[148] Dmitriyeva A.D., Filatov Y.V., Shalymov E.V., Venediktov V.Yu. Whispering gallery mode resonator as sensing element of microoptical gyro. Proceedings of the 2016 IEEE NW Russia Young Researchers in Electrical and Electronic Engineering Conference (EIConRusNW).

[149] Hall J.M.M., Shahraam Afshar V., Henderson M.R., François A., Reynolds T., Riesen N., Monro T.M. (2015). Method for predicting whispering gallery mode spectra of spherical microresonators. Opt. Express.

[150] Yao Y., Yao J., Kris Narasimhan V., Ruan Z., Xie C., Fan S., Cui Y. (2012). Broadband light management using low-Q whispering gallery modes in spherical nanoshells. Nat. Commun..

[151] Krupka J., Derzakowski K., Abramowicz A., Tobar M.E., Geyer R.G. (1999). Use of whispering-gallery modes for complex permittivity determinations of ultra-low-loss dielectric materials. IEEE Trans. Microw. Theory Tech..

[152] Lin N., Jiang L., Wang S., Yuan L., Xiao H., Lu Y., Tsai H. (2010). Ultrasensitive chemical sensors based on whispering gallery modes in a microsphere coated with zeolite. Appl. Opt..

[153] Zamora V., Díez A., Andrés M.V., Gimeno B. Chemical sensor applications of whispering-gallery modes resonances of thin capillaries with submicrometric wall. Proceedings of the SPIE Optical Sensors 2009.

[154] Lane S., Chan J., Thiessen T., Meldrum A. (2014). Whispering gallery mode structure and refractometric sensitivity of fluorescent capillary-type sensors. Sens. Actuators B Chem..

[155] Shao G.-H., Wu Z.-J., Chen J.-H., Xu F., Lu Y.-Q. (2013). Nonlinear frequency conversion of fields with orbital angular momentum using quasi-phase-matching. Phys. Rev. A.

[156] Barnett S.M., Allen L., Cameron R.P., Gilson C.R., Padgett M.J., Speirits F.C., Yao A.M. (2016). On the natures of the spin and orbital parts of optical angular momentum. J. Opt..

[157] Yao A.M., Padgett M.J. (2011). Orbital angular momentum: Origins, behavior and applications. Adv. Opt. Photonics.

[158] Marshall M.D., Lester M.I. (2005). Spectroscopic implications of partially quenched orbital angular momentum in the OH-water complex. J. Phys. Chem. B.

[159] Volke-Sepúlveda K., Chávez-Cerda S., Garcés-Chávez V., Dholakia K. (2004). Three-dimensional optical forces and transfer of orbital angular momentum from multiringed light beams to spherical microparticles. J. Opt. Soc. Am. B.

[160] Gorodetski Y., Shitrit N., Bretner I., Kleiner V., Hasman E. (2009). Observation of optical spin symmetry breaking in nanoapertures. Nano Lett..

[161] Yamashita S., Mita M., Fujita H., Yamamoto T., Kawai M., Yano M. Optical beam shaping by spatial light phase modulator with bidirectional tilt-piston micromirror array. Proceedings of the Conference on Lasers and Electro-Optics (CLEO).

[162] Hlady V., Pierce M., Pungor A. (1996). Novel method of measuring cantilever deflection during an AFM force measurement. Langmuir.

[163] Putman C.A.J., De Grooth B.G., Van Hulst N.F., Greve J. (1992). A theoretical comparison between interferometric and optical beam deflection technique for the measurement of cantilever displacement in AFM. Ultramicroscopy.

[164] Schaffera T.E., Hansma P.K. (1998). Characterization and optimization of the detection sensitivity of an atomic force microscope for small cantilevers. J. Appl. Phys..

[165] Vuong J., Ramantanis P., Frignac Y., Salsi M., Genevaux P., Bendimerad D.F., Charlet G. (2015). Mode coupling at connectors in mode-division multiplexed transmission over few-mode fiber. Opt. Express.

[166] Mecozzi A., Antonelli C., Shtaif M. (2012). Coupled Manakov equations in multimode fibers with strongly coupled groups of modes. Opt. Express.

[167] Ho K.-P., Kahn J.M. (2011). Mode-dependent loss and gain: Statistics and effect on mode-division multiplexing. Opt. Express.

[168] Pan Z., Weng Y., He X. Investigation of the nonlinearity in few mode fibers. Proceedings of the 13th International Conference on Optical Communications and Networks (ICOCN).

